# Dialkylboryl-Substituted Cyclic Disilenes Synthesized by Desilylation-Borylation of Trimethylsilyl-Substituted Disilenes

**DOI:** 10.3390/molecules26061632

**Published:** 2021-03-15

**Authors:** Kaho Tanaka, Naohiko Akasaka, Tomoyuki Kosai, Shunya Honda, Yuya Ushijima, Shintaro Ishida, Takeaki Iwamoto

**Affiliations:** Department of Chemistry, Graduate School of Science, Tohoku University, 6-3 Aramakiazaaoba, Aoba-ku, Sendai 980-8578, Japan; kcahhooir0738@gmail.com (K.T.); nao.akasaka1216@gmail.com (N.A.); t.cos243@gmail.com (T.K.); shunya.honda.p8@dc.tohoku.ac.jp (S.H.); yuya.ushijima.s4@dc.tohoku.ac.jp (Y.U.); ishida@tohoku.ac.jp (S.I.)

**Keywords:** borylation, desilylation, disilene, disilenide, trimethylsilyl, UV-vis spectrum, X-ray analysis

## Abstract

π-Electron systems of silicon have attracted attention because of their narrow HOMO-LUMO gap and high reactivity, but the structural diversity remains limited. Herein, new dialkylboryl-substituted disilenes were synthesized by the selective desilylation-borylation of the corresponding trimethylsilyl-substituted disilenes. The dialkylboryl-substituted disilenes were fully characterized by a combination of NMR spectroscopy, MS spectrometry, single-crystal X-ray diffraction analysis, and theoretical calculations. The longest-wavelength absorption bands of boryldisilenes were bathochromically shifted compared to the corresponding silyl-substituted disilenes, indicating a substantial conjugation between π(Si=Si) and vacant 2p(B) orbitals. In the presence of 4-(dimethylamino)pyridine (DMAP), the dialkylboryl groups in the boryl-substituted disilenes were easily converted to trimethylsilyl groups, suggesting the dialkylboryl-substituted disilenes in the presence of a base serve as the surrogates of disilenyl anions (disilenides).

## 1. Introduction

**Figure 1:**
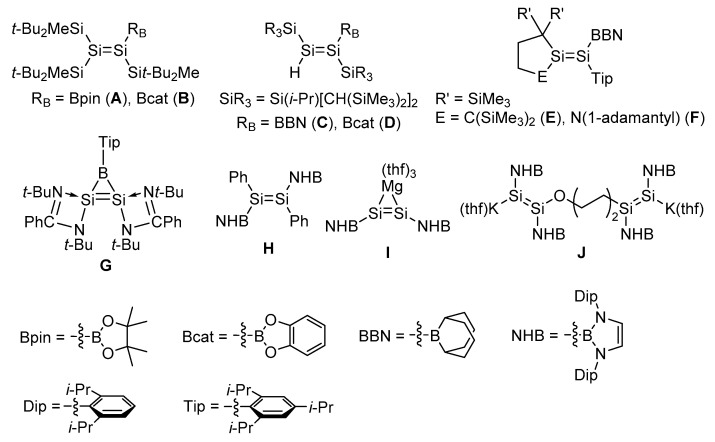
Reported isolable boryl-substituted disilenes.

Compounds with silicon-silicon double bonds (disilenes) have been extensively studied as π-electron systems of silicon with fascinating structural and electronic features arising from a higher-lying π orbital and a lower-lying π* orbitals compared to those of the corresponding alkenes [1,2,3,4,5,6,7,8,9]. Although disilenes inherently undergo auto-oligomerization due to their weaker π(Si=Si) bond energy compared to the corresponding σ(Si–Si) bond energy, the introduction of judiciously designed bulky protecting groups enables us to synthesize a variety of isolable disilenes that contain functional groups. Among these, boryl-substituted disilenes have been less investigated compared with other functionalized disilenes (Figure 1), although the boryldisilenes are anticipated to exhibit the structure and reactivity due to the conjugation between the π(Si=Si) and 2p(B) orbitals and the coordination of a base to the boryl group should alter the electronic structure and reactivity. Sekiguchi et al. have shown the synthesis of boryldisilenes **A**–**D** [10,11,12] and the substantial interaction between the 9-borabicyclo[3.3.1]nonyl (BBN) group and the Si=Si double bond in **C**. Our group has synthesized BBN-substituted disilenes **E** and **F** [13,14] and found a bathochromically-shifted absorption band due to the substantial push-pull effects in disilene **F** and the activation of H_2_ molecule via the cleavage of the Si–B and Si=Si bonds. Very recently, Roesky et al. have reported boradisilacycle **G** having 2π aromatic character [15]. Cui et al. have reported new disilene **H** [16] and disilenides **I** and **J** substituted by *N*-heterocyclic boryl (NHB) groups [17].

**Scheme 1:**
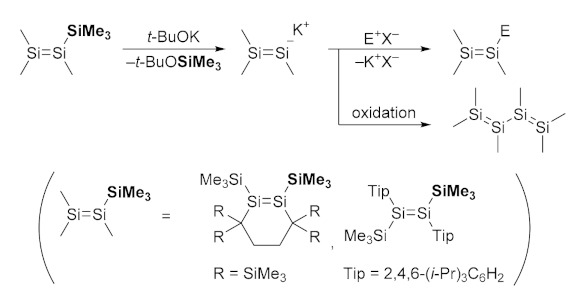
Selective desilylation and subsequent reactions of trimethylsilyl-substituted disilenes.

Recently, we have developed a mild and convenient method to generate a disilenide from the corresponding stable trimethylsilyl-substituted disilene via selective cleavage of Si(sp^2^)-Si(sp^3^) bond (desilylation) (Scheme 1). This method does not require a harsh reaction condition, such as the reduction with alkali metals, which have been often used for the synthesis of disilenes and disilenides (disilicon analogs of vinyl anion). This method enables us to obtain new disilenes with polycyclic aromatic hydrocarbon (PAH) groups, tetra-1,3-siladienes, and a tetrasila-1,3-dienide (a silicon analog of, but-1,3-dienyl anion) [18,19]. These results prompted us to examine the synthesis of new disilenes with other functional groups. Herein, we report the synthesis of new dialkylboryl-substituted disilenes via the desilylation-borylation of trimethylsilyl-substituted disilenes, as well as their molecular structures and some reactions. Substantial interaction between the Si=Si double bond and the dialkylboryl groups was revealed by the UV-vis spectra and TD-DFT calculations. The unprecedented substitution reactions of boryldisilenes in the presence of a base were also reported.

## 2. Results and Discussion

### 2.1. Desilylation-Borylation of Silyl-Substituted Disilenes

**Scheme 2:**
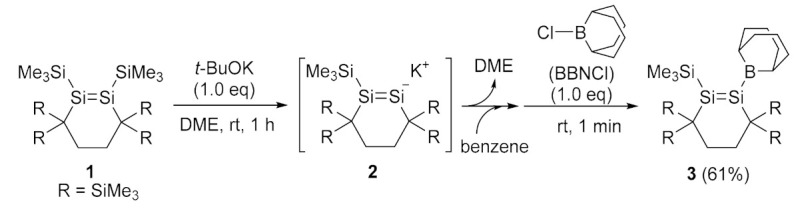
Synthesis of (dialkylboryl)disilacyclohexene **3**.

**Scheme 3:**
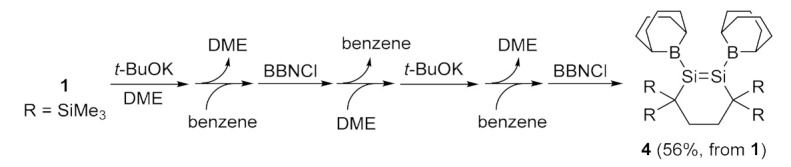
Synthesis of 1,2-di(dialkylboryl)disilacyclohexene **4**.

Monoborylated disilene **3** was obtained by the following desilylation-borylation reaction (Scheme 1). Treatment of 1,2-bis(trimethylsilyl)-1,2-disilacyclohexene **1** and one equivalent of *t*-BuOK in 1,2-dimethoxyethane (DME) lead to the quantitative formation of the corresponding disilenide **2 [19]**, which was confirmed by ^1^H NMR spectroscopy. After the volatiles was removed in vacuo, the resulting residue was dissolved in dry benzene. As soon as one equivalent of 9-chloro-9-borabicyclo[3.3.1]nonane (BBNCl) was added to the benzene solution, the color of the solution turned from orange to red. After the resulting insoluble materials were filtered off and the volatiles was removed in vacuo, recrystallization from toluene provided analytically pure boryldisilene **3** as reddish-orange crystals in 61% yield (Scheme 2). Similarly, 1,2-diboryldisilene **4** was obtained as purple crystals in 56% yield from **1** by the double desilylation-borylation reactions without the isolation of **3** (Scheme 3). Notably, the desilylation by *t*-BuOK in the second step occurred selectively, although boryldisilene **3** has an electron-deficient dialkylboryl substituent as well as a Si=Si double bond. Borylsilanes are known to react with a base, such as an alkyllithium, *N*-heterocyclic carbenes, *t*-BuOK, etc. to provide the corresponding silyl anion and/or silylborate [20,21,22,23,24], while *t*-BuOK can add across the Si=Si double bond in (Me_3_Si)(*i*-Pr_3_Si)Si=Si(SiMe_3_)(Si*i*-Pr_3_) to provide the corresponding disilanyl anion [25]. The selective formation of **4** should be due to severe steric demand of a combination of the dialkylboryl group and *t*-BuO^−^ moiety to prohibit the coordination of *t*-BuO^−^ to the boron atom, as the 4-(*N*,*N*-dimethylamino)pyridine (DMAP), which is a base with a more planar structure than *t*-BuO^−^, can coordinate the boron atom (vide infra). The structures of **3** and **4** were determined by a combination of NMR spectroscopy, MS spectrometry, elemental analyses, and single-crystal X-ray diffraction (XRD) analysis.

### 2.2. X-ray Analysis of **3** and **4**

**Figure 2:**
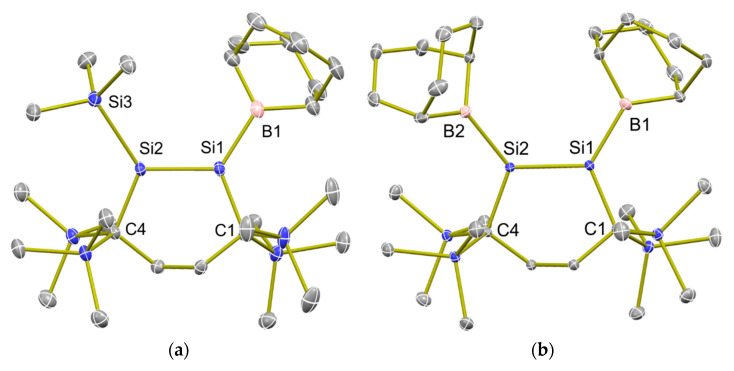
Molecular structures determined by single-crystal XRD analysis (atomic displacement parameters set at 50% probability; hydrogen atoms omitted for clarity). (**a**) Compound **3** and (**b**) compound **4**.

Molecular structures of **3** and **4** determined by single-crystal XRD analysis are shown in Figure 2, and the selected metric parameters of **3** and **4,** as well as the related compounds, are shown in Table 1. The geometries around the Si=Si double bonds in disilenes **3** and **4** are slightly *trans*-bent [the *trans*-bent angles *θ*: 9.8° [=*Si*(BBN)] and 9.6° (=*Si*SiMe_3_) as well as 4.4° and 8.0° [=*Si*(BBN)] ] and twisted [the twist angles *τ*(Si=Si) 19.3° for **3** and 16.7° for **4**]. The Si=Si distances [2.1990(8) Å and 2.2114(5) Å for **3** and **4**, respectively] are longer than that of **1** [2.1762(5) Å] with the increase of the number of dialkylboryl groups. The Si–B distances [1.994(3) Å for **3**; 1.9851(14) Å and 2.0156(15) Å for **4**] fall into the range of the Si–B distances of the reported boryl-substituted disilenes [1.945–2.022 Å] [10,11,13,14,17]. The twist angle of the Si–B bond, *τ*(Si–B), which is defined as the angle between the axis that bisects the Si(sp^2^)–Si(sp^2^)–C(SiMe_3_)_2_ angle and the axis that bisects the C–B–C angle as viewed along the Si(sp^2^)–B axis (Table 1) and should qualitatively represent the dihedral angle of the 3p orbital on the double bond silicon atom and the 2p orbital on the boron atom, is 5.0°: the orientation of the boryl group in **3** is suitable for the conjugation between the π(Si=Si) orbital and 2p(B) orbital. The angles *τ*(Si–B) for **4** [15.5°(Si1) and 41.2°(Si2)] are larger than that of **3** (5.0°), which would be attributed to the severe steric demand of two dialkylboryl groups.

### 2.3. UV-Vis Spectra of ***3*** and ***4***

**Figure 3:**
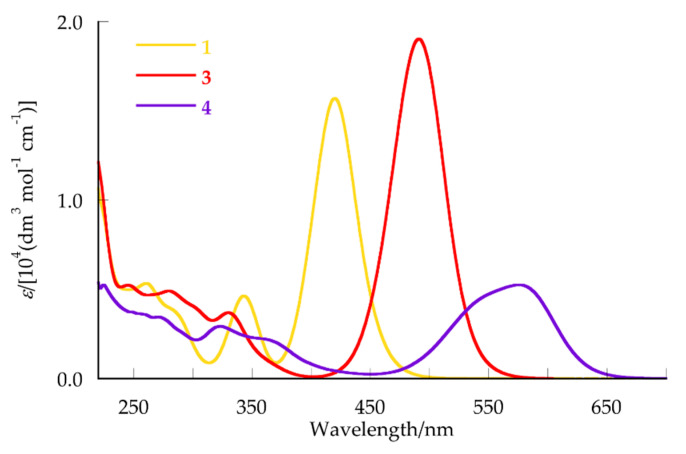
UV-vis spectra of disilenes **3** (reddish-orange), **4** (purple), and **1** (yellow) in hexane at room temperature.

Each disilene **3** and **4** exhibits a distinct absorption band in the visible region (Figure 3). The longest-wavelength absorption band maximum (*λ*_max_) of **3** (491 nm) is bathochromically shifted by 71 nm relative to that of **1** (420 nm) and close to that of BBN and silyl-substituted disilene **C** (469 nm) [11]. Conversely, the band shape of **4** in the visible region is different from that of **3**. Disilene **4** exhibits an absorption band at 576 nm with a shoulder around 540 nm. The absorption band maximum of **4** is far bathochromically shifted compared to that of monoboryldisilene **3**. The substantial bathochromic shift of the absorption band with the increase in the number of the dialkylboryl group suggests the effective conjugation between the Si=Si double bond and the vacant 2p orbital(s) of the boryl group(s).

**Figure 4:**
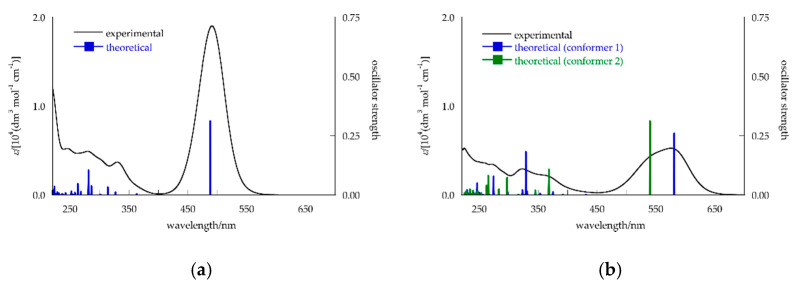
UV-vis spectra in hexane at room temperature and theoretical band positions and oscillator strengths (vertical bars) calculated at the B3LYP-D3/B1(hexane)//B3PW91-D3/6-31G(d) level of theory (B1: 6-311G(2df) [Si], 6-311G(d) [N, C, B, H]). (**a**) Compound **3** and (**b**) compound **4**.

**Figure 5:**
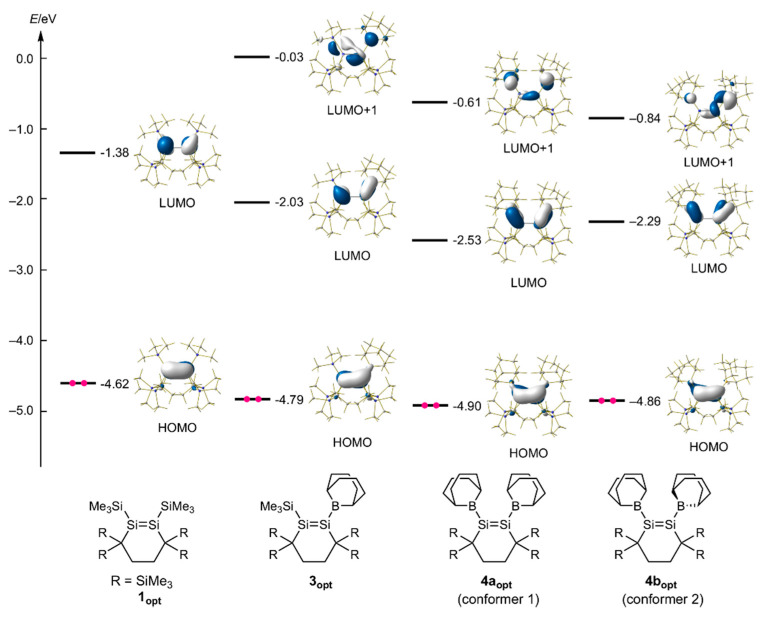
Frontier Kohn–Sham orbitals and their energy levels of **1_opt_**, **3_opt_, 4a_opt_**, and **4b_opt_** calculated at the B3LYP-D3/B1 level of theory (B1: 6-311G(2df) [Si], 6-311G(d) [N, C, B, H]) (R = SiMe_3_).

The electronic structures of **3** and **4** were further examined by density functional theory (DFT) calculations. The structural characteristics of **3** optimized at the B3PW91-D3/6-31G(d) level of theory (**3_opt_**) are in good agreement with that obtained by XRD analysis of **3** (Table 2). The band positions and oscillator strengths of **3_opt_** determined by TD-DFT calculations at the B3LYP/B1 level of theory (basis B1: 6-311G(2df) [Si], 6-311G(d) [other atoms]) were also consistent with those obtained from the experimental absorption spectra (Figure 4a), suggesting that the structure of **3** in solution is close to those observed in the single crystals. The HOMO of **3_opt_** consists predominantly of an in-phase combination of the π(Si=Si) orbital and 2p(B) orbital, while the LUMO and LUMO+1 consist of an in-phase and out-of-phase combination of the π*(Si=Si) orbital and 2p(B) orbital, respectively (Figure 5). Both the HOMO [−4.79 eV] and LUMO [−2.03 eV] of **3_opt_** are lower in energy than those of **1_opt_** [−4.62 and −1.38 eV] due to the efficient interaction between the Si=Si double bond and the boryl group. The HOMO-LUMO gap of **3_opt_** [2.76 eV] is smaller than that of **1_opt_** [3.24 eV], suggesting that the 2p(B) orbital should interact with the π*(Si=Si) orbital more effectively than the π(Si=Si) orbital. A comparison of the experimental and theoretical spectra disclosed that the longest-wavelength absorption band of **3** (491 nm) should be attributed to the HOMO→LUMO transition. The substantial bathochromic shift of the absorption band in **3** relative to **1** should be explained by the smaller HOMO-LUMO gap resulting from the effective interaction between the Si=Si double bond and the boryl group. In the case of **4**, two conformers, conformer 1 (**4a_opt_**) and conformer 2 (**4b_opt_**), were optimized as local minima. The major structural difference between **4a_opt_** and **4b_opt_** is in the orientation of the BBN group relative to the Si=Si double bond. The twist angles *τ*(Si–B), which represents the overlap between the 3p orbital on the double bond Si atom and the 2p orbital on the boron atom, are 8.3° and 8.1 for **4a_opt_**, while those are 13.2 and 42.6 for **4b_opt_**, which resemble those of **4.** The geometry around the Si–B bond in **4a_opt_** is more suitable for the conjugation between the Si=Si double bond and 2p orbital on the B atom than that of **4b_opt_**. Although the HOMO and LUMO of **4a_opt_** and **4b_opt_** are π(Si=Si) and π*(Si=Si) orbitals with a substantial contribution of 2p(B) orbitals of two boryl groups, the HOMO and LUMO levels of **4a_opt_** [−4.90 and −2.53 eV] are lower than those of **4b_opt_** [−4.86 and −2.29 eV], which is consistent with the smaller twist angles *τ*(Si–B) for **4a_opt_** compared to those of **4b_opt_**. Compound **4a_opt_** is more stable by only 0.34 kJ mol^−1^ in free energy (298.15 K) than **4b_opt_**, implying that both conformers can contribute to the UV-vis spectrum of **4** in solution. As expected, the observed absorption spectrum of **4** is consistent with the combined absorption bands of **4a_opt_** and **4b_opt_** obtained from the TD-DFT calculations (Figure 4b). The longest-wavelength absorption band of **4** (576 nm) should be attributed to the HOMO→LUMO transition of **4a_opt,_** and its shoulder peak (~540 nm) should be due to the HOMO→LUMO transition of **4b_opt_**. The observed UV-vis spectra of boryldisilenes **3** and **4** revealed that the BBN group effectively interacts with the Si=Si double bond, which is consistent with the previous reports on BBN-substituted disilenes [11,13,14].

### 2.4. Reactions of Boryldisilenes with 4-(N,N-Dimethylamino)pyridine (DMAP)

**Scheme 4:**
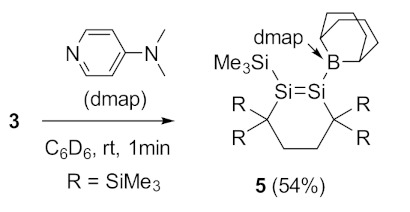
Reaction of **3** and 4-(*N*,*N*-dimethylamino)pyridine (DMAP).

**Figure 6:**
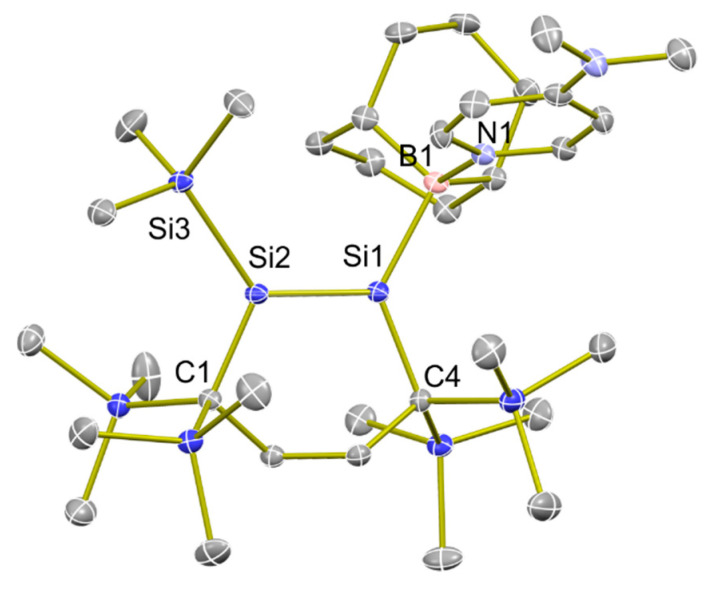
Preliminary XRD analysis of **5** (atomic displacement parameters set at 50% probability; hydrogen atoms omitted for clarity).

Boryldisilene **3** reacted immediately with a typical Lewis base, 4-(*N*,*N*-dimethylamino)pyridine (DMAP) at room temperature to provide DMAP-adduct **5** in 54% yield as orange crystals (Scheme 4). The structure of **5** was identified by a combination of multinuclear NMR spectra, MS spectrometry, and the preliminary single-crystal XRD analysis (Figure 6). Although it was difficult to obtain high-quality single crystals suitable for discussion of the structural parameters, the XRD analysis unequivocally revealed that DMAP coordinates to the boron atom in the solid state. DMAP coordinating to the boryl group of **5** was easily removed by BPh_3_: treatment of **5** with BPh_3_ in C_6_D_6_ provided boryldisilene **3** almost quantitatively.

**Figure 7:**
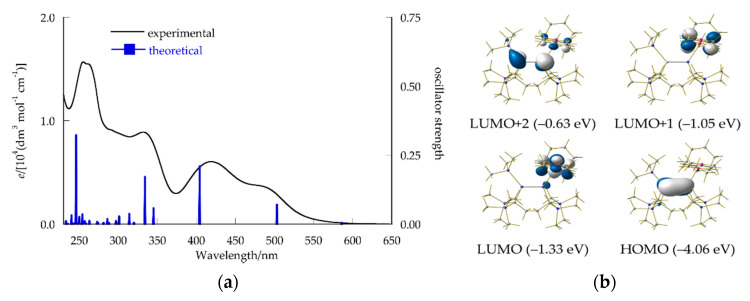
(**a**) UV-vis absorption spectrum of **5** in hexane at room temperature and theoretical band positions and oscillator strengths (vertical bars) calculated at the B3LYP-D3/B1(hexane)//B3PW91-D3/6-31G(d) level of theory (B1: 6-311G(2df) [Si], 6-311G(d) [N, C, B, H]). (**b**) Selected frontier Kohn–Sham orbitals calculated at the same level.

The UV-vis spectrum of **5** disclosed that the coordination of DMAP to the boryl group affects the electronic structure of the Si=Si double bond. A hexane solution of **5** exhibited its longest wavelength absorption band at 418 nm with a shoulder band around 500 nm (Figure 7a). A comparison of experimental and theoretical UV-vis spectra predicted by TD-DFT calculations discloses that the absorption band at 418 and the shoulder peak around 500 nm would be attributed to a π(Si=Si)→π*(Si=Si) transition and a π(Si=Si)→π*(DMAP), respectively (Figure 7b). The π(Si=Si)→π*(Si=Si) transition band of **5** (416 nm) is hypsochromically shifted relative to that of boryldisilene **3** (491 nm) and comparable to that of **1** (420 nm) [19], indicating the lack of the vacant 2p orbital on the boron atom resulting from the coordination of DMAP.

In the ^29^Si NMR spectrum, two ^29^Si resonances due to the unsaturated silicon nuclei [87.9 (=*Si*SiMe_3_) and 195.2 (=Si(BBN·DMAP) ppm] were observed for **5**. Upon coordination of DMAP to the boryl moiety on the Si=Si double bond, the chemical shift (δSi) for the boryl-substituted double bond silicon nuclei (195.2 ppm) is substantially downfield shifted compared to that of **3** (128.7 ppm). Conversely, the δSi for the SiMe_3_-substituted double bond silicon nuclei (87.9 ppm) is upfield-shifted compared to that of **3** (187.2). The substantial difference in the chemical shifts of the double bond silicon nuclei was reproduced qualitatively by the GIAO calculations of **5_opt_** [73.1 (=*Si*SiMe_3_) and 237.7 (=Si(BBN·DMAP) ppm] and **3_opt_** [209.1 (=*Si*SiMe_3_) and 136.2 (=Si(BBN) ppm] at the B97-D3/def2-TZVP [26] level of theory (Table S4). As Strohman, Kaupp et al. have theoretically shown that the large difference in δSi of the double bond Si nuclei in the unsymmetrically substituted disilenes is mainly due to the spatial extent and orientation of the occupied and unoccupied molecular orbitals around the Si=Si double bond [27], the remarkable change in the δSi of the double bond Si nuclei upon coordination of DMAP is consistent with the lack of the conjugation between the π(Si=Si) and 2p(B) orbitals.

**Scheme 5:**
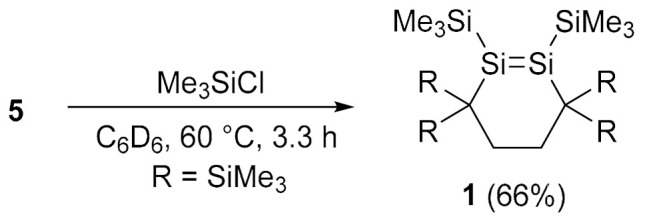
Reaction of **5** with Me_3_SiCl.

**Scheme 6:**
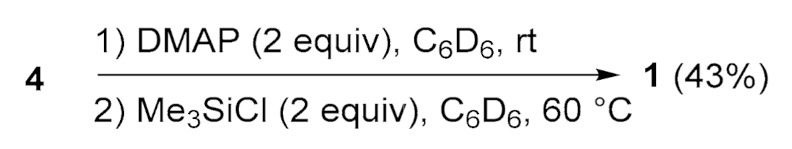
Reaction of **4** with DMAP (2 equiv) followed by Me_3_SiCl (2 equiv).

Notably, treatment of **5** with Me_3_SiCl in benzene-*d*_6_ at 60 °C for 3 h provided disilene **1** in 66% yield (Scheme 5), indicating that the DMAP-coordinated boryl group on the double bond silicon atom was substituted by SiMe_3_ group. In this reaction, **5** formally serves as a disilenide like **2**. As the reaction of boryldisilene **3** and Me_3_SiCl does not proceed in benzene-*d*_6_ at 60 °C, the coordination of DMAP to the boryl group is crucial for this substitution reaction, similar to the reactions of pinacolboryl-substituted silanes, which can work as silyl nucleophiles in the presence of a base [20,21,22,23,24]. Similar substitution reaction occurred upon treatment of diboryldisilene **4** with DMAP (2 equiv) followed by Me_3_SiCl (Scheme 6) to provide **1** in 43% in two steps, although the characterization of the intermediates of this reaction has been unsuccessful due to the unresolved broad NMR signals. The observed reactions suggest a boryl-substituted disilene can be a synthetic equivalent of a disilenyl anion (a disilenide) in the presence of a Lewis base.

## 3. Conclusions

We successfully synthesized dialkylboryl-substituted cyclic disilenes **3** and **4** by the desilylation-borylation of the corresponding 1,2-bis(trimethylsilyl)-substituted cyclic disilene **1**. The effective conjugation between the Si=Si double bond and the dialkylboryl group is reflected in a substantial bathochromic shift of the longest-wavelength absorption band. 4-(*N*,*N*-Dimethylamino)pyridine (DMAP) coordinates to the boryl group of monoboryldisilene **3** to afford the corresponding boranuidyldisilene **5**. The DMAP-coordinated boryl group in **5** was converted to the Me_3_Si group after treatment with Me_3_SiCl. In the presence of 2 equiv of DMAP, diboryldisilene **4** also undergoes a similar substitution reaction to afford 1,2-bis(trimethylsilyl)disilene **1**. These results imply that boryl-substituted disilenes have the potential to serve as surrogates of disilenides in the presence of an appropriate base.

## 4. Materials and Methods

### 4.1. General Procedure

All reactions treating air-sensitive compounds were performed under argon or nitrogen atmosphere using a high-vacuum line, standard Schlenk techniques, or a glove box, as well as dry and oxygen-free solvents. ^1^H (500 MHz), ^11^B (160 MHz), ^13^C (125 MHz), and ^29^Si (99 MHz) NMR spectra were recorded on a Bruker Avance III 500 FT NMR spectrometer. The ^1^H NMR chemical shifts in benzene-*d*_6_ (C_6_D_6_) were referenced to the residual C_6_D_5_H signal (δ 7.16). The ^13^C{^1^H} and ^29^Si{^1^H} NMR chemical shifts were relative to Me_4_Si (δ 0.00). The ^11^B NMR chemical shifts were relative to BF_3_·OEt_2_ (δ 0.00). Sampling of air-sensitive compounds was carried out using a VAC NEXUS 100027-type glove box. Mass spectra were recorded on a Bruker Daltonics SolariX 9.4T or a JEOL JMS-Q1050 spectrometer. UV-vis spectra were recorded on a JASCO V-660 spectrometer.

### 4.2. Materials

Benzene, hexane, tetrahydrofuran (THF), and toluene were dried by using a VAC (Vacuum Atmospheres Company) solvent purifier 103991. 1,2-Dimethoxyethane (DME) and toluene-*d*_8_ were dried over LiAlH_4_ or potassium mirror and then distilled prior to use through a vacuum line. Benzene-*d*_6_ was degassed and dried over molecular sieves (4Å). Disilene **1** [19] and 9-chloro-9-borabicyclo[3.3.1]nonane (BBNCl) [28] were prepared according to the procedure described in the literature. Chlorotrimethylsilane, 4-(*N*,*N*-dimethylamino)pyridine (DMAP), and potassium *t*-butoxide (*t*-BuOK) were purchased from commercial sources and used without further purification.

### 4.3. Synthesis of (Dialkylboryl)disilene ***3***

In a Schlenk tube (50 mL) equipped with a magnetic stir bar, disilene **1** (134 mg, 2.45 × 10^−1^ mmol), *t*-BuOK (28.9 mg, 2.58 × 10^−1^ mmol) and DME (9.0 mL) was placed. After stirring the mixture for 1 h at room temperature, disilenide **2** was formed as a sole product, which was confirmed by ^1^H NMR spectroscopy. Then DME was removed in vacuo; the resulting residue was dissolved in benzene (12 mL). To the solution, a benzene solution (3.0 mL) of 9-chloro-9-borabicyclo[3.3.1]nonane (BBNCl) (38.4 mg, 2.45 × 10^−1^ mmol) was added, and the mixture was stirred for 1 min at room temperature. The resulting insoluble materials were filtered off and washed with benzene. The volatiles was removed from the filtrate in vacuo. Recrystallization from hexane at –35 °C provided boryldisilene **3** as reddish-orange crystals (89.0 mg, 1.50 × 10^−1^ mmol) in 61% yield. Single crystals suitable for X-ray diffraction analysis were obtained by recrystallization from toluene at room temperature.

**3**: reddish-orange crystals; mp. 103 °C (decomp); ^1^H NMR (C_6_D_6_, 500 MHz, 295 K) δ 0.21 (s, 9H, Si(C*H*_3_)_3_), 0.29 (s, 9H, Si(C*H*_3_)_3_), 0.34 (s, 9H, Si(C*H*_3_)_3_), 0.38 (s, 9H, Si(C*H*_3_)_3_), 0.48 (s, 9H, Si(C*H*_3_)_3_), 1.41–1.49 (m, 2H, BBN), 1.90–2.11 (m, 12H, BBN + C*H*_2_), 2.49 (brs, 2H, BBN), 2.52–2.60 (m, 2H, C*H*_2_); ^13^C NMR (C_6_D_6_, 125 MHz, 296 K) δ 1.0 (Si(*C*H_3_)_3_), 1.2 (Si(*C*H_3_)_3_), 3.9 (Si(*C*H_3_)_3_), 4.29 (Si(*C*H_3_)_3_), 4.34 (Si(*C*H_3_)_3_), 20.7 (*C*), 23.7 (*C*H_2_), 26.6 (*C*), 33.5 (2 × *C*H), 34.5 (*C*H_2_), 34.69 (*C*H_2_), 34.72 (*C*H_2_), 35.6 (*C*H_2_); ^29^Si NMR (C_6_D_6_, 99 MHz, 295 K) δ –10.0 (*Si*Me_3_), 0.2 (*Si*Me_3_), 1.1 (*Si*Me_3_), 1.6 (*Si*Me_3_), 1.8 (*Si*Me_3_), 128.7 (=*Si*BBN), 187.2 [=*Si*SiMe_3_]; ^11^B NMR (C_6_D_6_, 160 MHz, 295 K) δ 92.1; UV-vis (hexane) *λ*_max_/nm (*ε*) 490 (1.9 × 10^4^); MS (EI, 70 eV) *m*/*z* (%) 594 (3.8, M^+^), 521 (7.0, M^+^–SiMe_3_); Anal. Calcd for C_27_H_63_BSi_7_: C, 54.48; H, 10.67%. Found: C, 54.30; H, 10.62%.

### 4.4. Synthesis of 1,2-Di(dialkylboryl)disilene ***4***

In a Schlenk tube (50 mL) equipped with a magnetic stir bar, disilene **1** (249 mg, 4.56 × 10^−1^ mmol), *t*-BuOK (51.6 mg, 4.60 × 10^−1^ mmol), and DME (30 mL) were placed. After stirring for 1 h at room temperature, disilenide **2** was formed as a sole product, which was confirmed by ^1^H NMR spectroscopy. Then DME was removed in vacuo; the resulting residue was dissolved in benzene (29 mL). To the solution, a benzene solution (0.5 mL) of 9-chloro-9-borabicyclo[3.3.1]nonane (BBNCl) (72.1 mg, 4.61 × 10^−1^ mmol) was added, and the mixture was stirred for 1 min at room temperature. Then the volatiles was removed in vacuo; the resulting residue was dissolved in DME (12 mL). To the solution, *t*-BuOK (51.1 mg, 4.55 × 10^−1^ mmol) was added, and the mixture was stirred for 1 min at room temperature. Then the volatiles was removed in vacuo; the resulting residue was dissolved in benzene (14 mL) again. To the solution, a benzene solution (0.5 mL) of BBNCl (71.2 mg, 4.55 × 10^−1^ mmol) was added, and the mixture was stirred for 1 min at room temperature. The resulting insoluble materials were filtered off and washed with benzene, and then the volatiles was removed from the filtrate in vacuo. The formation of 1,2-diboryldisilene **4** as a major product was confirmed by the ^1^H NMR spectrum. Recrystallization from hexane at –35 °C gave **4** as purple crystals (164 mg, 2.55 × 10^−1^ mmol) in 56% yield. Singles crystal suitable for X-ray diffraction analysis were obtained by recrystallization from toluene at room temperature.

**4**: purple crystals; mp. 106 °C (decomp); ^1^H NMR (C_6_D_6_, 500 MHz, 297 K) δ 0.27 (s, 18H, Si(C*H*_3_)_3_), 0.39 (s, 18H, Si(C*H*_3_)_3_), 1.38–1.45 (m, 4H, BBN), 1.96–2.11 (m, 22H, BBN, overlapping with C*H*_2_C*H*_2_), 2.57–2.63 (m, 6H, BBN, overlapping with C*H*_2_C*H*_2_); ^13^C NMR (C_6_D_6_, 125 MHz, 297 K) δ 1.4 (Si(*C*H_3_)_3_), 4.5 (Si(*C*H_3_)_3_), 23.6 (*C*H_2_), 25.3 (*C*), 34.5 (*C*H_2_), 34.86 (*C*H_2_), 34.94 (two BBN-*C*H), 35.3 (*C*H_2_); ^29^Si NMR (C_6_D_6_, 99 MHz, 297 K) δ 0.5 (*Si*Me_3_), 2.0 (*Si*Me_3_), 166.2 (*Si*=*Si*), ^11^B NMR (C_6_D_6_, 160 MHz, 296 K) δ 93.7; UV-vis (hexane) *λ*_max_/nm (ε) 576 (5.3 × 10^3^), 550 (sh); MS (EI, 70 eV) *m*/*z* (%); 642 (2.0, M^+^), 569 (1.1, M^+^–SiMe_3_); Anal. Calcd for C_32_H_68_B_2_Si_6_: C, 59.77; H, 10.66%. Found: C, 59.41; H, 10.80%.

### 4.5. Synthesis of Boryldisilene-DMAP Complex ***5***

In a J. Young NMR tube, boryldisilene **3** (43.3 mg, 7.27 × 10^−2^ mmol), 4-(*N*,*N*-dimethylamino)pyridine (10.4 mg, 8.51 × 10^−2^ mmol), and C_6_D_6_ (0.7 mL) were placed at room temperature. The color of the solution gradually turned from red to orange. After 1 min, the formation of disilene **5** as a sole product was confirmed by ^1^H NMR spectroscopy. Then C_6_D_6_ was removed in vacuo. After recrystallization from hexane at –35 °C gave **5** as orange crystals (18.1 mg, 2.52 × 10^−2^ mmol) in 54% yield. Single crystals of **5** suitable for X-ray diffraction analysis were obtained by recrystallization from toluene at room temperature.

**5**: orange crystals; mp. 134 °C; ^1^H NMR (C_6_D_6_, 500 MHz, 298 K) δ 0.18 (br, 9H, Si(C*H*_3_)_3_), 0.41 (s, 9H, Si(C*H*_3_)_3_), 0.45 (s, 18H, Si(C*H*_3_)_3_), 0.76 (s, 9H, Si(C*H*_3_)_3_), 1.55–1.58 (m, 1H, BBN), 1.66 (brs, 1H, BBN), 1.78–2.03 (m, 14H, BBN overlapping with N(C*H*_3_)_2_), 2.11–2.29 (m, 6H, BBN overlapping with CH_2_CH*H*), 2.55–2.73 (m, 3H, BBN overlapping with CH_2_CH*H*), 5.84 (brs, 2H, C*H* of DMAP), 8.51 (brs, 2H, C*H* of DMAP); ^13^C NMR (toluene-*d*_8_, 125 MHz, 233 K) δ 1.9 (Si(*C*H_3_)_3_), 2.1 (Si(*C*H_3_)_3_), 4.9 (Si(*C*H_3_)_3_), 5.2 (Si(*C*H_3_)_3_), 16.0 (*C*), 23.1 (*C*H), 24.7 (*C*H_2_), 25.6 (*C*H_2_), 27.4 (*C*), 29.8 (*C*H_2_), 30.0 (*C*H_2_), 34.4 (*C*H_2_), 35.2 (*C*H_2_), 35.6 (*C*H_2_), 36.8 (*C*H_2_), 38.0 (*C*H_3_ of DMAP), 38.2 (*C*H), 105.4 (*C*H of DMAP), 146.5 (*C*H of DMAP), 153.6 (*C* of DMAP); ^29^Si NMR (toluene-*d*_8_, 99 MHz, 233 K) δ –14.0 (*Si*(CH_3_)_3_), –2.1 (*Si*(CH_3_)_3_), 0.8 (*Si*(CH_3_)_3_), 1.3 (*Si*(CH_3_)_3_), 2.8 (*Si*(CH_3_)_3_), 87.9 (=*Si*SiMe_3_), 195.2 (=*Si*(BBN·DMAP)); ^11^B NMR (C_6_D_6_, 160 MHz, 296 K) δ 2.0; UV-vis (hexane) *λ*_max_/nm (ε) 418 (6.0 × 10^3^), 489 (sh); MS (EI, 70 eV) *m*/*z* (%) 716 (2.5, M^+^), 643 (12, M^+^–SiMe_3_); Anal. Calcd for C_34_H_73_BN_2_Si_7_: C, 56.93; H, 10.26; N, 3.91%. Found: C, 57.23; H, 10.41; N, 4.03%.

### 4.6. Reaction of ***5*** with Triphenylborane

In a J. Young NMR tube, **3** (8.2 mg, 1.4 × 10^−2^ mmol), DMAP (2.6 mg, 2.1 × 10^−2^ mmol), and C_6_D_6_ (0.5 mL) were charged. After the formation of **5** was confirmed by a ^1^H NMR spectrum, BPh_3_ (5.7 mg, 2.4 × 10^−2^ mmol) was added to the mixture. The quantitative formation of **3** was confirmed by the ^1^H NMR spectrum.

### 4.7. Reaction of ***5*** with Me_3_SiCl

In a J. Young NMR tube, **5** (21.5 mg, 3.0 × 10^−2^ mmol), C_6_D_6_ (0.5 mL) and Me_3_SiCl (3.3 mg, 3.0 × 10^−2^ mmol) were placed for 3.3 h at 60 °C, and then Mes*H (11.5 mg, 4.67 × 10^−2^ mmol) was added as an internal standard. The formation of disilene **1** (NMR yield 66%) was confirmed by the ^1^H NMR and ^29^Si-^1^H 2D HMBC NMR spectra. A byproduct was a complex of 9-chloro-9-borabicyclo[3.3.1]nonane and DMAP (**6**), which was confirmed by the comparison of the NMR spectra with those of the authentic sample obtained alternatively from 9-chloro-9-borabicyclo[3.3.1]nonane and DMAP as follows: In a J. Young NMR tube, 9-chloro-9-borabicyclo[3.3.1]nonane (10.5 mg, 67.1 μmol), DMAP (8.6 mg, 70.4 μmol) and C_6_D_6_ (0.5 mL) were charged at room temperature. Removal of the volatiles and recrystallization from hexane at −25 °C gave **6** as a white powder (17.5 mg, 62.8 μmol) in 94% yield.

**6**: a white powder; mp. 137–140 °C (decomp); ^1^H NMR (C_6_D_6_, 500 MHz, 295 K) δ 1.58–1.63 (m, 1H, BBN), 1.68–1.76 (m, 4H, BBN), 1.94 (s, 6H, C*H*_3_), 2.12–2.20 (m, 3H, BBN), 2.24–2.42 (m, 4H, BBN), 3.18–3.22 (m, 2H, BBN), 5.54 (d, *J* = 7.5 Hz, 2H, aryl), 8.02 (d, *J* = 7.5 Hz, 2H, aryl); ^13^C NMR (C_6_D_6_, 125 MHz, 296 K) δ 25.1 (*C*H_2_, BBN), 25.4 (*C*H_2_, BBN), 31.9 (*C*H_2_, BBN), 32.8 (*C*H_2_, BBN), 38.4 (*C*H_3_), 106.7 (DMAP), 143.7 (DMAP); ^11^B NMR (C_6_D_6_, 160 MHz, 296 K) δ 7.4 (BBN); MS (EI, 70 eV) *m*/*z* (%); 156 (7.1, M^+^–dmap), 121 (100, M^+^–BBNCl-H); Anal. Calcd for C_15_H_24_BClN_2_: C, 64.66; H, 8.68; N, 10.05%. Found: C, 64.46; H, 8.70; N, 10.05%. The ^13^C resonance due to methine carbon nuclei next to the boron nuclei was not observed.

### 4.8. Reaction of Diboryldislene ***4*** with DMAP Followed by Me_3_SiCl

In a J. Young NMR tube, diboryldisilene **4** (19.4 mg, 3.0 × 10^−2^ mmol), DMAP (8.2 mg, 6.71 × 10^−2^ mmol), and C_6_D_6_ (0.5 mL) were placed at room temperature. The color of the solution gradually turned from violet-blue of **4** to pink–purple. Then, Me_3_SiCl (6.56 mg, 6.04 × 10^−2^ mmol) was added to the reaction mixture at room temperature and placed for 3 h at 60 °C. Mes*H (11.4 mg, 4.63 × 10^−2^ mmol) was added as an internal standard. The formation of disilene **1** (NMR yield: 43%) was confirmed by the ^1^H and ^29^Si-^1^H 2D HMBC NMR spectra.

### 4.9. Single-Crystal X-ray Diffraction Analysis

Single crystals of **3**, **4**, and **5** suitable for X-ray diffraction analysis were obtained by recrystallization from toluene at room temperature. For data collection, single crystals coated by Apiezon^®^ grease were mounted on thin glass fiber and transferred into the cold nitrogen gas stream of the diffractometer. X-ray diffraction data were collected on a Bruker AXS APEX II CCD diffractometer using graphite-monochromated Mo-Kα radiation. An empirical absorption correction based on the multiple measurements of equivalent reflections was applied using the program SADABS [29], and the structure was solved by direct methods and refined by full-matrix least-squares against *F*^2^ using all data (SHELX-2014 or 2018) [30]. The molecular structure was analyzed using the Yadokari-XG software [31]. CCDC-2062117 to 2062119 contain the supplementary crystallographic data for this paper. These data can be obtained free of charge via http://www.ccdc.cam.aC.uk/conts/retrieving.html accessed on 2 February 2021 (or from the CCDC, 12 Union Road, Cambridge CB2 1EZ, UK; Fax: +44 1223 336033; E-mail: deposit@ccdc.cam.aC.uk).

Crystal Data for **3** (CCDC-2062117): C_27_H_63_BSi_7_ (M = 595.21 g/mol), triclinic, space group *P*-1 (no. 2), *a* = 9.2894(4) Å, *b* = 12.3390(5) Å, *c* = 17.6712(7) Å, α = 76.8100(10)°, *β* = 88.0070(10)°, *γ* = 70.2510(10)°, *V* = 1854.10(13) Å^3^, *Z* = 2, *T* = 100(2) K, *μ*(MoKα) = 0.273 mm^−1^, *D*_calc_ = 1.066 g/cm^3^, 27816 reflections measured (3.604° ≤ 2*θ* ≤ 54.996°), 8501 unique (*R*_int_ = 0.0595, *R*_sigma_ = 0.0624), which were used in all calculations. The final *R*1, w*R*2, and GOF were 0.0462 (*I* > 2σ(*I*)), 0.1073 (all data), and 1.017, respectively.

Crystal Data for **4** (CCDC-2062118): C_32_H_68_B_2_Si_6_ (M = 643.02 g/mol), triclinic, space group *P*-1 (no. 2), *a* = 12.3914(3) Å, *b* = 12.7560(3) Å, *c* = 13.8222(3) Å, α = 110.7350(10)°, *β* = 106.9280(10)°, *γ* = 93.0890(10)°, *V* = 1924.43(8) Å^3^, *Z* = 2, *T* = 100(2) K, *μ*(MoKα) = 0.273 mm^−1^, *D*_calc_ = 1.110 g/cm^3^, 27,816 reflections measured (3.340° ≤ 2*θ* ≤ 55.000°), 8836 unique (*R*_int_ = 0.0255, *R*_sigma_ = 0.0253), which were used in all calculations. The final *R*1, w*R*2, and GOF were 0.0293 (*I* > 2σ(*I*)), 0.0799 (all data), and 1.017, respectively.

Crystal Data for **5** (preliminary) (CCDC 2062119): C_34_H_73_BN_2_Si_7_ (M = 717.38 g/mol), orthorhombic, space group *P*2_1_2_1_2_1_ (no. 19), *a* = 10.1552(8) Å, *b* = 20.8205(14) Å, *c* = 20.8919(15) Å, *V* = 4417.3(6) Å^3^, *Z* = 4, *T* = 100(2) K, *μ*(MoKα) = 0.273 mm^−1^, *D*_calc_ = 1.079 g/cm^3^, 26017 reflections measured (3.900° ≤ 2*θ* ≤ 55.00°), 8420 unique (*R*_int_ = 0.0255, *R*_sigma_ = 0.0253), which were used in all calculations. Absolute structure parameter was 0.01(6). The final *R*1, w*R*2, and GOF were 0.0422 (*I* > 2σ(*I*)), 0.0883 (all data), and 0.950, respectively.

### 4.10. Computational Study

Theoretical calculations were performed essentially using the Gaussian 09 [32] or GRRM14 [33,34] programs. Geometry optimizations and frequency analyses of **3**, **4**, and **5** were carried out at the B3PW91-D3/6-31G(d) level of theory. Imaginary frequencies were not encountered in any of the optimized structures. The supplemental file “optimized_structures.xyz” contains the calculated Cartesian coordinates and energies of all molecules reported in this study. The transition energies and oscillator strengths of the electron transition in hexane (Tables S5≤S8) were calculated using a time-dependent hybrid DFT method (TD-DFT) at the B3LYP-D3/B1(hexane)//B3PW91-D3/6-31G(d) level of theory (basis B1: 6-311G(2df) [Si], 6-311G(d) [N, C, B, H]). NMR chemical shifts (Table S4) were calculated at the GIAO/B97-D3/def2-TZVP(benzene)//B3PW91-D3/6-31G(d) level of theory [26]. The natural bond orbital (NBO) analysis was performed at the B3PW91-D3/6-31G(d) level of theory using the NBO 7.0 program [35] (Table S3).

## Figures and Tables

**Table 1:**
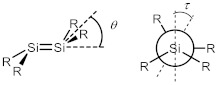
Selected structural parameters of **3**, **4**, and related compounds.

Compound	Distance/Å		Bent Angle *θ*/° (Angle Sum/°)		Twist Angle *τ*/°	
	Si=Si	Si–B	Si1	Si2	Si=Si	Si–B
**3**	2.1990(8)	1.994(3)	9.8 (*Si*BBN) (359.15(8))	9.6 (*Si*SiMe_3_) (359.16(6))	19.3	5.0
**4**	2.2114(5)	1.9851(14) (Si1) 2.0156(15) (Si2)	4.4 (359.83(5))	8.0 (359.36(5))	16.7	15.5 (Si1) 41.2 (Si2)
**1**	2.1762(5)	–	12.9 (358.52(3))	6.5 (359.61(3))	17.7	–

**Table 2:** Selected structural parameters of **3**, **4**, and **5** calculated at the B3PW91-D3/6-31G(d) level of theory.

Compound	Distance/Å		Bent Angle *θ*/° (Angle Sum/°)		Twist Angle τ/°	
	Si=Si	Si–B	Si1	Si2	Si=Si	Si–B
**3_opt_**	2.19225	1.97039	11.8 (358.80)	10.3 (359.10)	20.2	2.5
**4a_opt_**	2.20152	1.98758 (Si1) 1.99036 (Si2)	11.7 (358.80)	15.0 (358.04)	27.7.	8.1 (Si1) 8.3 (Si2)
**4b_opt_**	2.20285	1.98078(Si1) 2.00371 (Si2)	1.8 (359.97)	7.1 (359.50)	18.9	13.2 (Si1) 42.6 (Si2)
**5_opt_**	2.19009	2.10907	10.1 (359.14)	5.4 (359.69)	8.1	–

## Data Availability

The data reported in this study are available in the Supplementary Materials.

## Supplementary Materials

The following are available online. NMR spectra of compounds **3**, **4**, and **5,** as well as the details of theoretical study.
