# Supplementary material for: Dialkylboryl-Substituted Cyclic Disilenes Synthesized by Desilylation-Borylation of Trimethylsilyl-Substituted Disilenes
Source: Molecules. 2021 Mar 15;26(6):1632. doi: 10.3390/molecules26061632 (PMC8001088; doi:10.3390/molecules26061632)
Supplement: Supplementary file 1 [file molecules-26-01632-s001.pdf]

Supplementary Materials

Dialkylboryl-Substituted Cyclic Disilenes Synthesized by Desilylation-Borylation of Trimethylsilyl-Substituted Disilenes

Kaho Tanaka, Naohiko Akasaka, Tomoyuki Kosai, Shunya Honda, Yuya Ushijima, Shintaro Ishida, and Takeaki Iwamoto*

Department of Chemistry, Graduate School of Science, Tohoku University, 6-3 Aramakiazaaoba, Aoba-ku, Sendai 980-8578, Japan
Correspondence: takeaki.iwamoto@tohoku.ac.jp; Tel.: +81-22-795-6558 (T.I.)

Contents

1. NMR Spectra S2
  Boryldisilene **3** S2
  Diboryldisilene **4** S5
  DMAP-coordinated Boryldisilene **5** S8
  Reaction of **5** and BPh_3 .... S11
  Reaction of **5** and Me_3SiCl S12
  Reaction of **4** and DMAP Followed by Me_3SiCl S14
  Reaction of DMAP and BBNCl S16
2. Details of Theoretical Study S20
  Optimized Structures of **3**, **4**, and **5** S20
  Natural Bond Orbital (NBO) Analysis S21
  GIAO Calculations S22
  TD-DFT Calculations S22
3. Temperature-Dependent UV-vis Spectrum of **4** S30

1. NMR Spectra

Boryldisilene 3

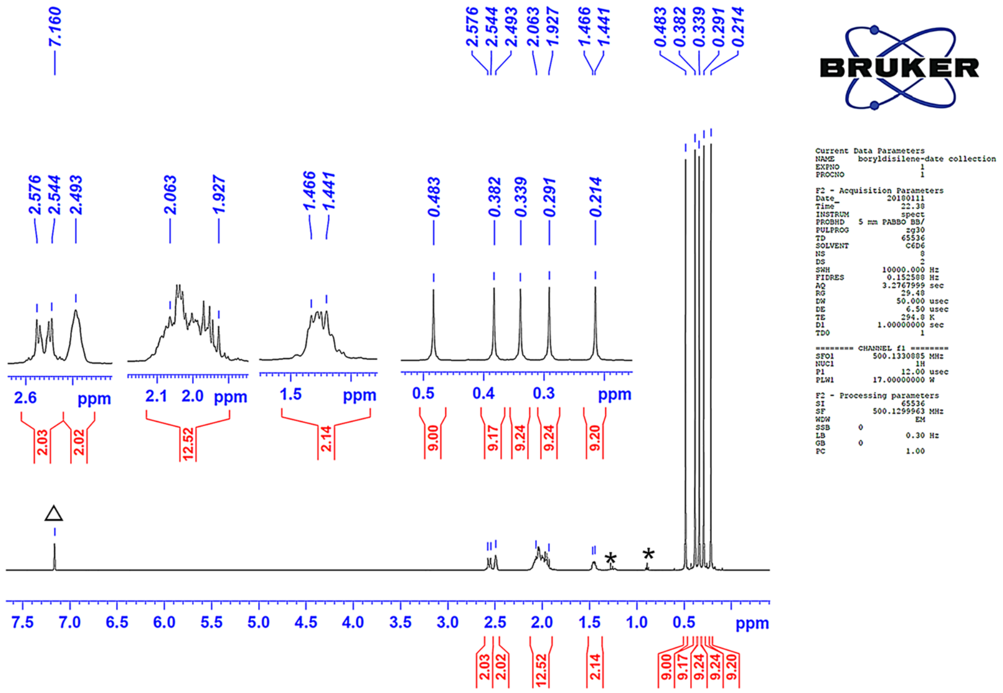

Figure S1. ^{1}H NMR spectrum of **3** in C_6D_6 at 295 K (△= C_6D_5H, ＊= hexane).

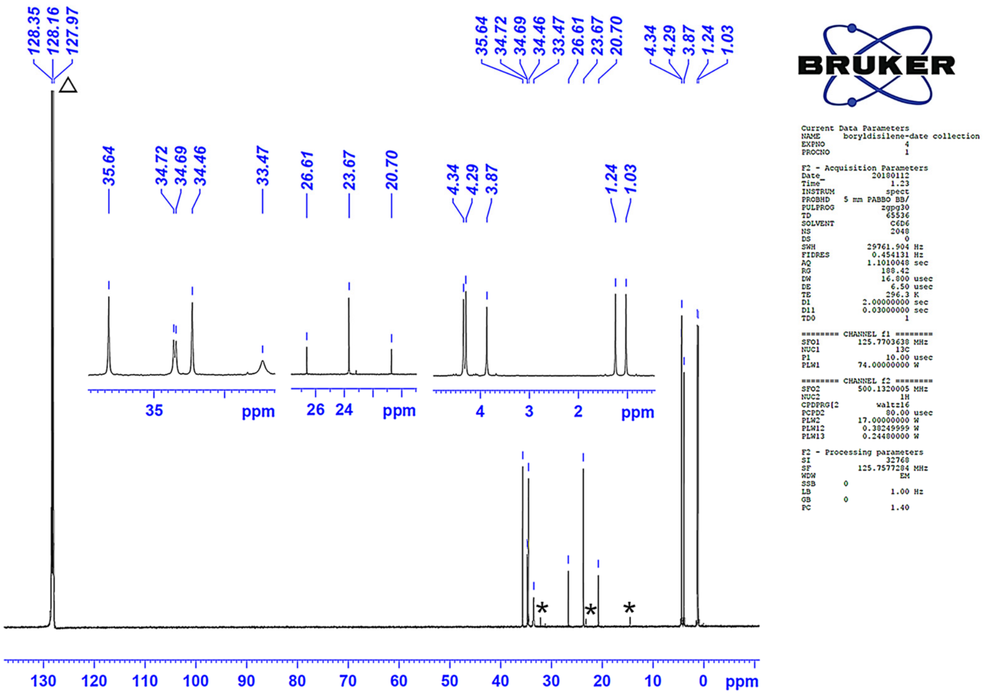

Figure S2. ^{13}C{^{1}H} NMR spectrum of **3** in C_6D_6 at 296 K (△= C_6D_6,, ＊= hexane).

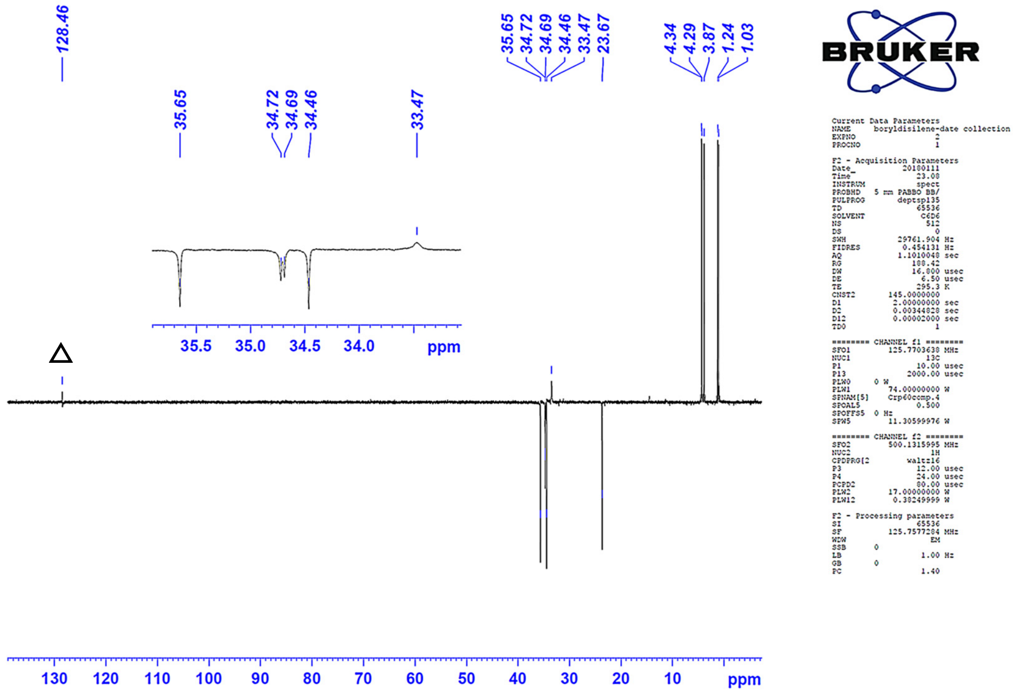

Figure S3. ${}^{13}C\{{}^{1}H\}$ NMR spectrum of **3** using DEPT 135 pulse sequence in C_6D_6 at 295 K.

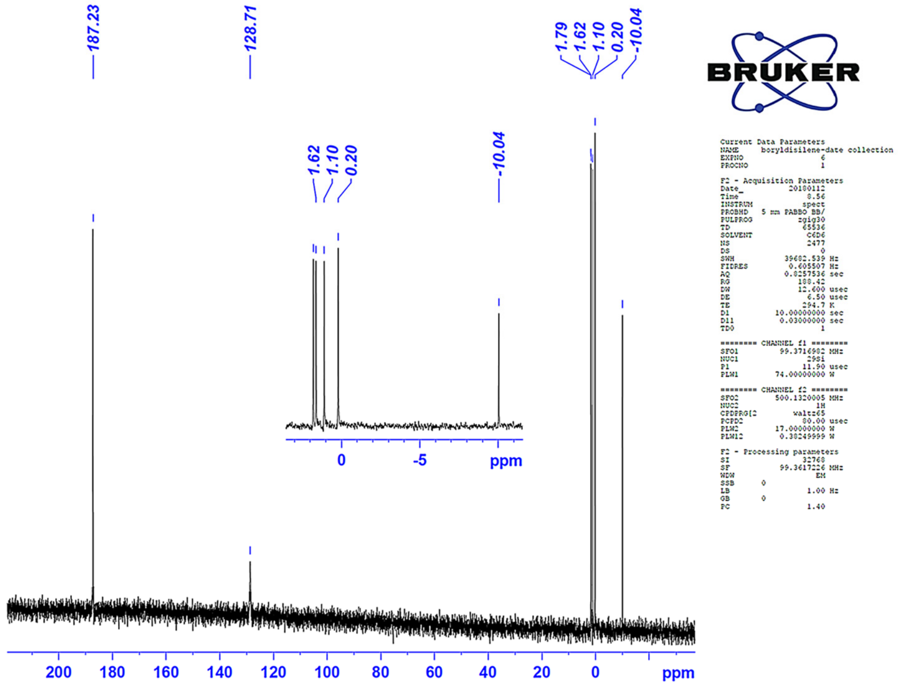

Figure S4. ${}^{29}Si\{{}^{1}H\}$ NMR spectrum of **3** using the inverse-gated pulse sequence in C_6D_6 at 295 K.

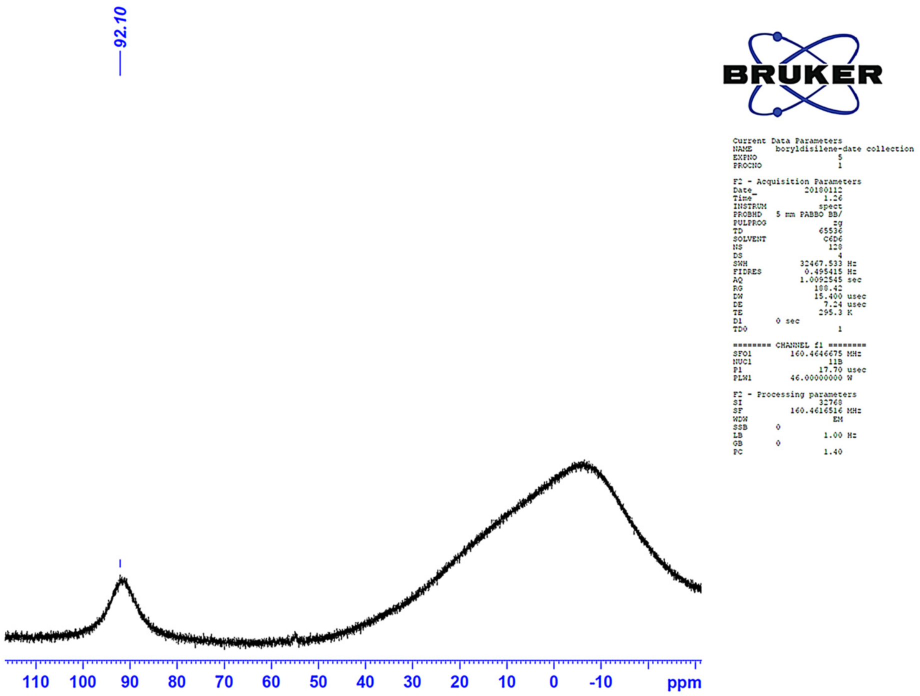

Figure S5. ^{11}B NMR spectrum of **3** in C_6D_6 at 295 K.

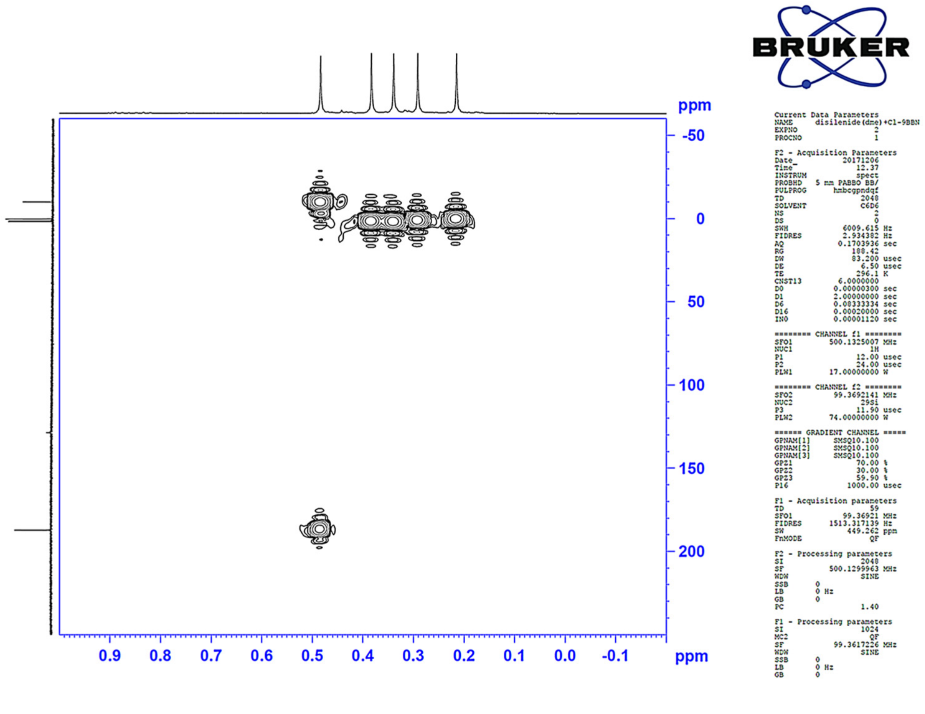

Figure S6. ^{29}Si-^{1}H 2D HMBC NMR spectrum of **3** in C_6D_6 at 296 K.

Diboryldisilene 4

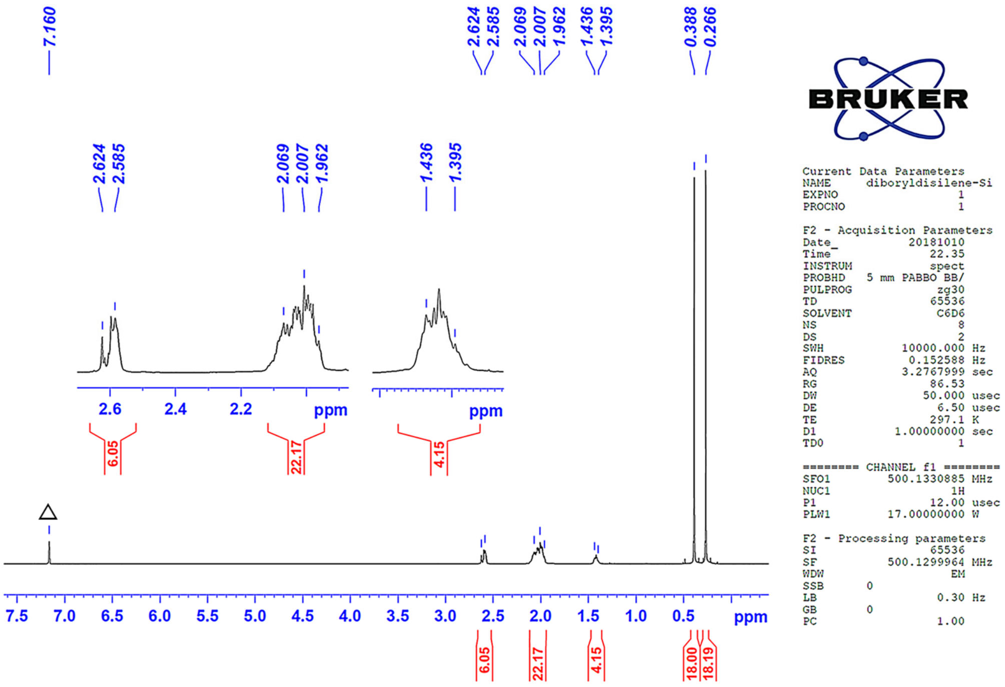

Figure S7. ^{1}H NMR spectrum of **4** in C_6D_6 at 297 K (△= C_6D_5H).

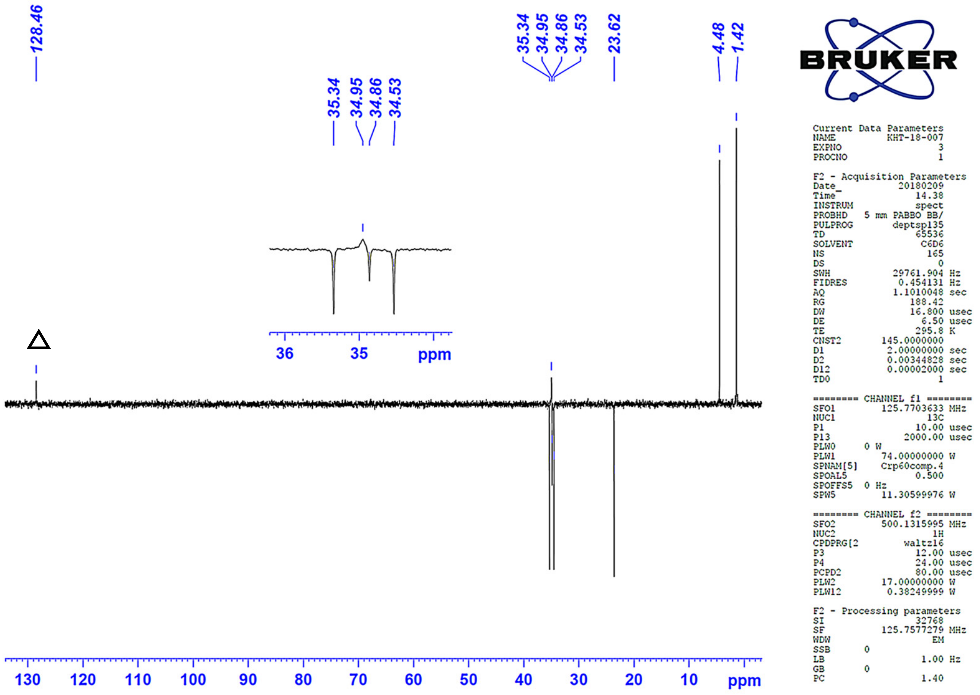

Figure S8. $^{13}C\{^{1}H\}$ NMR spectrum of **4** using DEPT 135 pulse sequence in C_6D_6 at 296 K (△= C_6D_6).

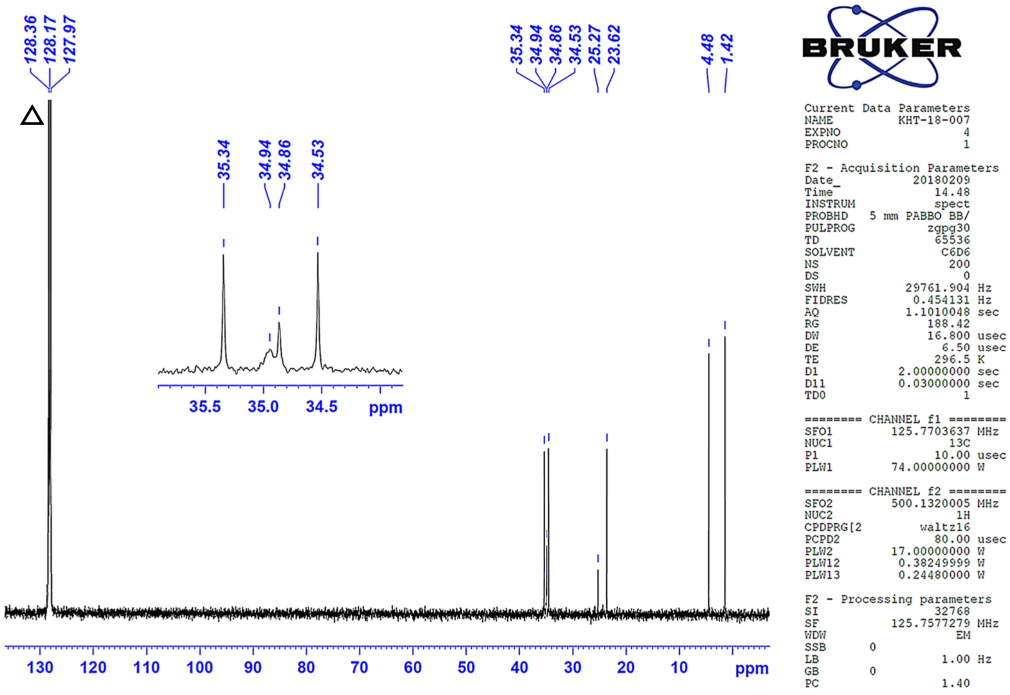

Figure S9. $^{13}C\{^1H\}$ NMR spectrum of **4** in C_6D_6 at 297 K (△= C_6D_6).

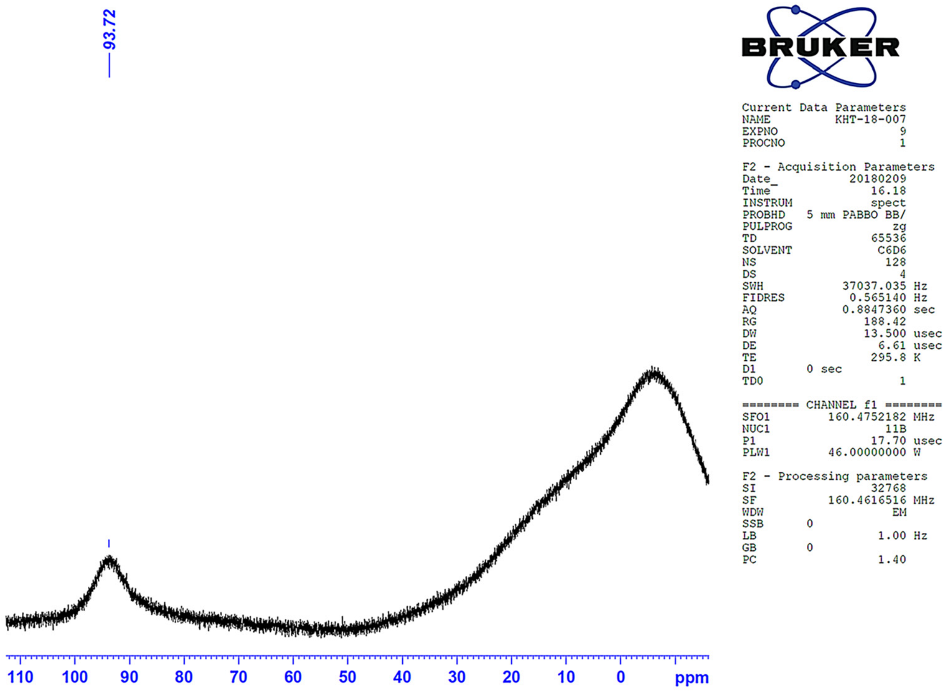

Figure S10. ^{11}B NMR spectrum of **4** in C_6D_6 at 296 K.

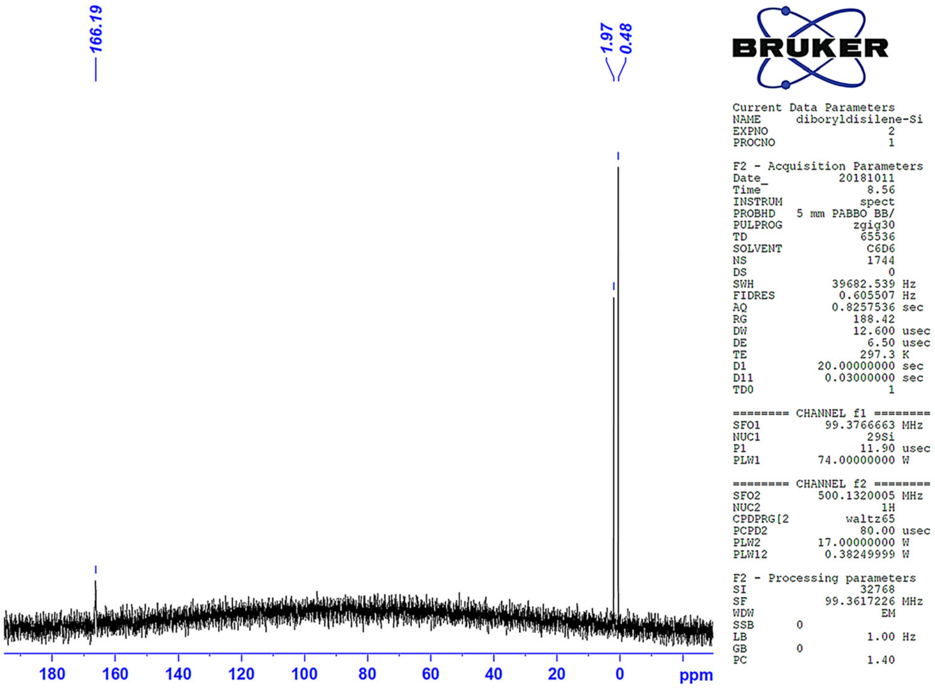

Figure S11. $^{29}Si\{^1H\}$ NMR spectrum of **4** using the inverse-gated pulse sequence in C_6D_6 at 297 K.

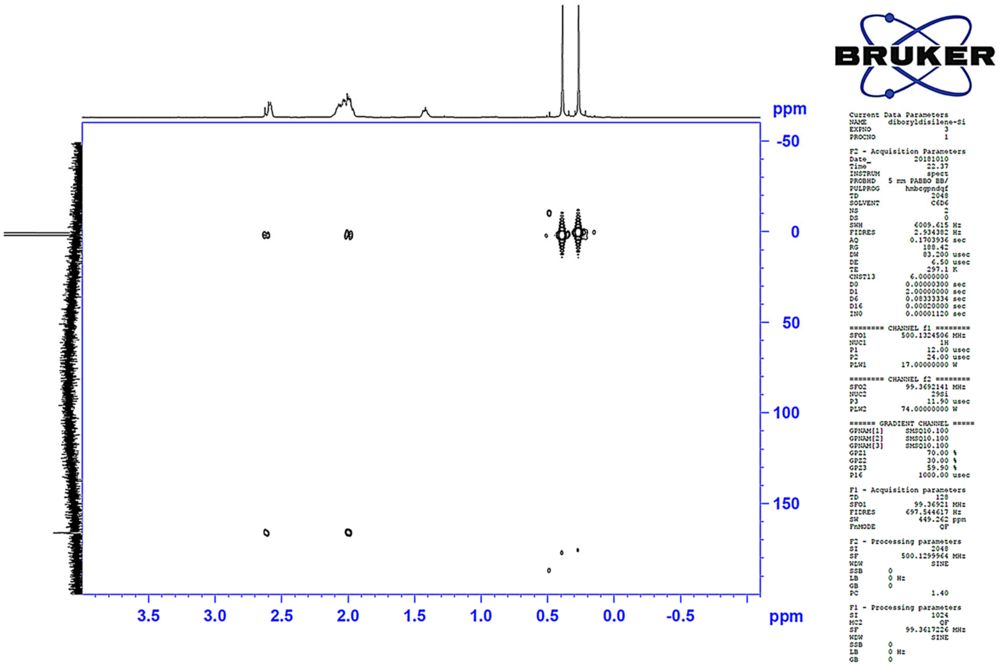

Figure S12. ^{29}Si-^1H 2D HMBC NMR spectrum of **4** in C_6D_6 at 297 K.

DMAP-coordinated Boryldisilene 5

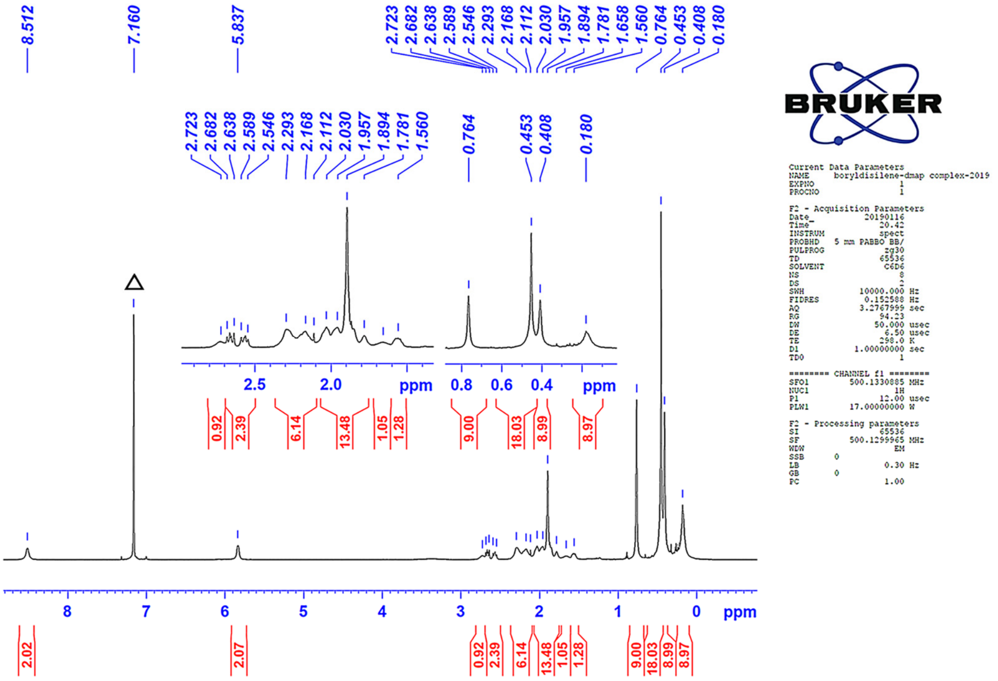

Figure S13. ^{1}H NMR spectrum of **5** in C_6D_6 at 298 K (△= C_6D_5H).

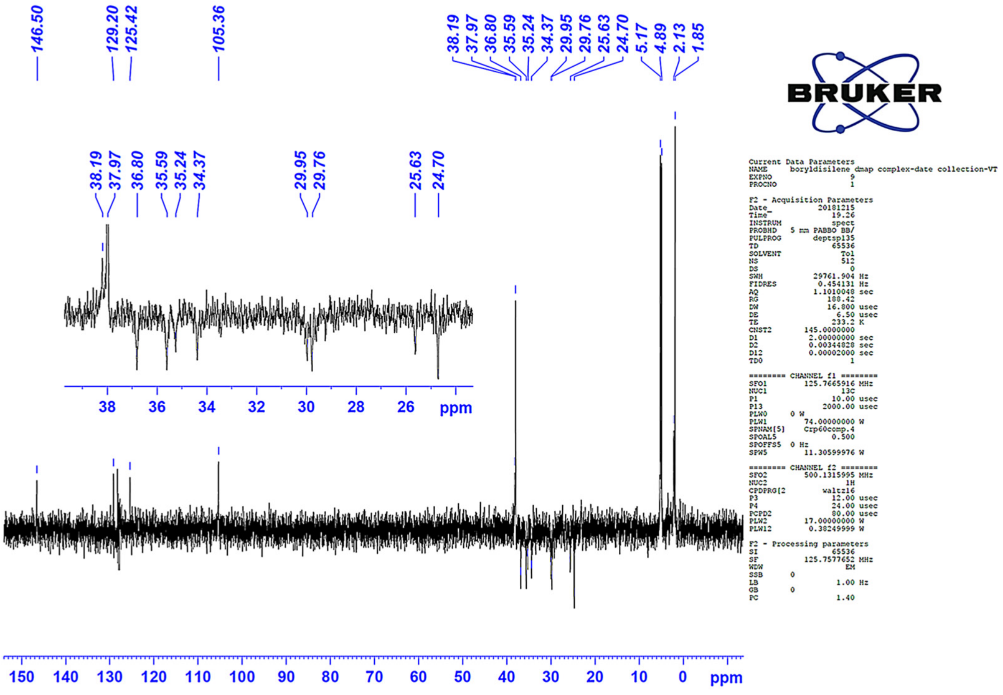

Figure S14. $^{13}C\{^{1}H\}$ NMR spectrum of **5** using DEPT 135 pulse sequence in toluene-d_8 at –40°C.

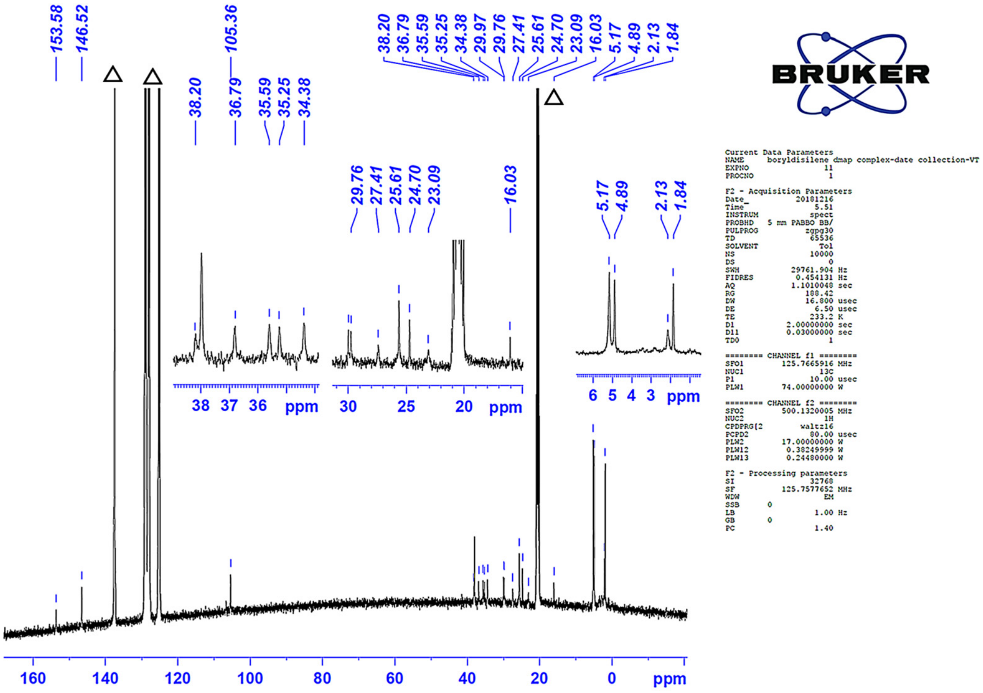

Figure S15. $^{13}C\{^{1}H\}$ NMR spectrum of **5** in toluene-d_8 at –40°C (△= toluene-d_8).

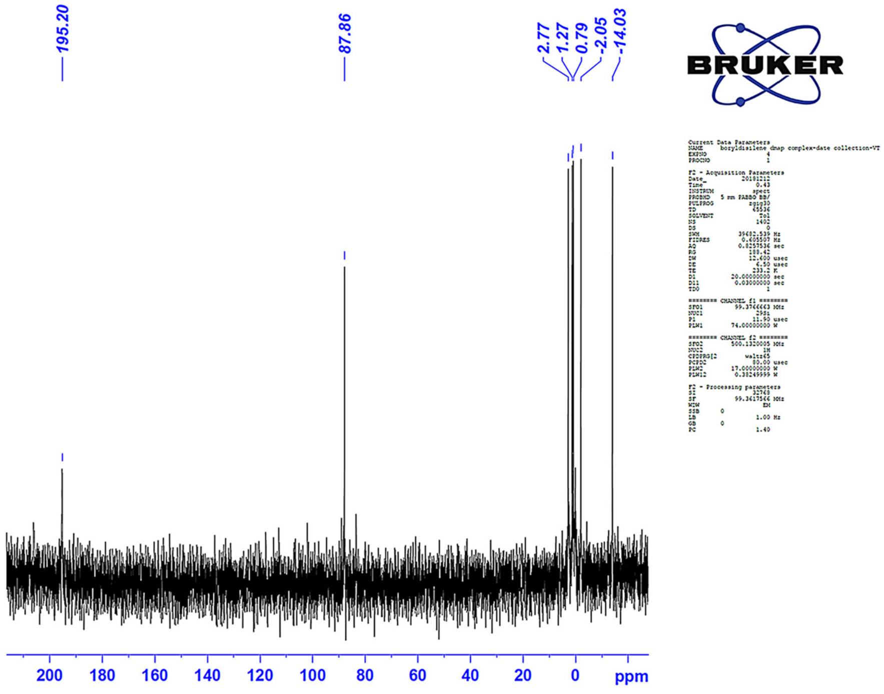

Figure S16. $^{29}Si\{^{1}H\}$ NMR spectrum of **5** in toluene-d_8 at –40 °C.

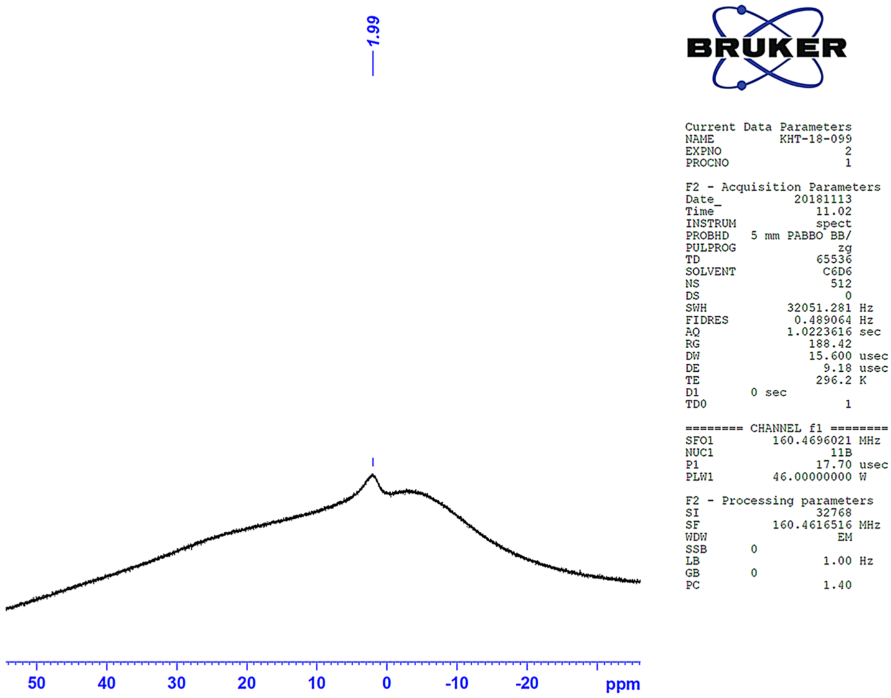

Figure S17. ^{11}B NMR spectrum of **5** in C_6D_6 at 296 K.

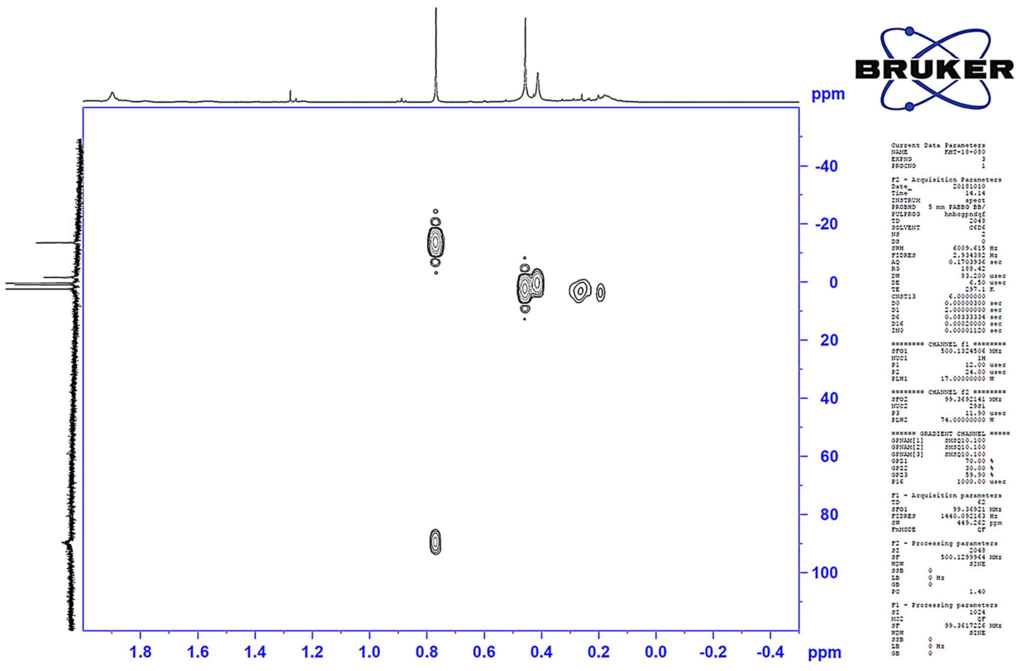

Figure S18. ^{29}Si-^{1}H 2D HMBC NMR spectrum of **5** in C_6D_6 at 297 K.

Reaction of 5 and BPh_3

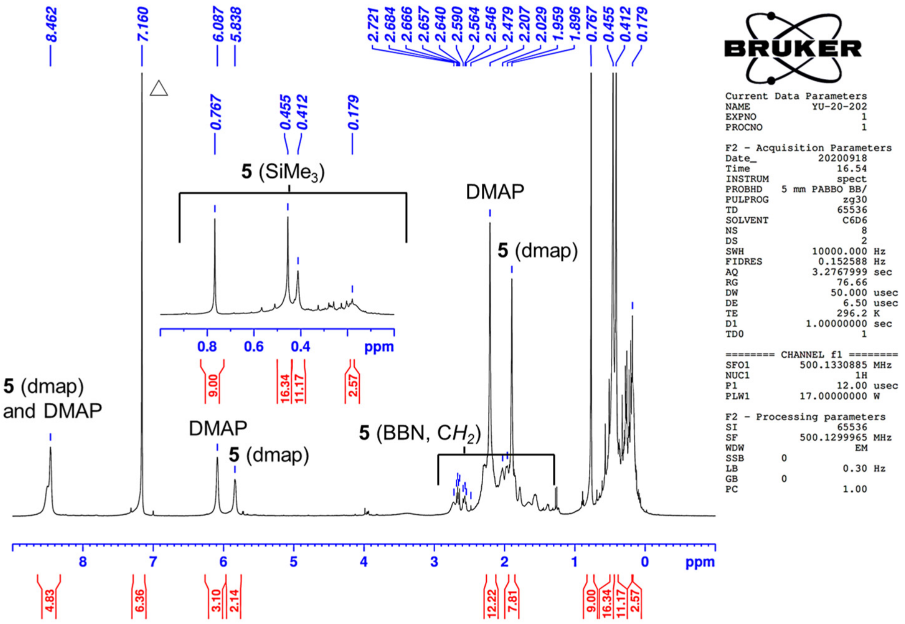

Figure S19. ^1H NMR spectrum of DMAP adduct **5** in C_6D_6 at 296 K (△= C_6D_5H).

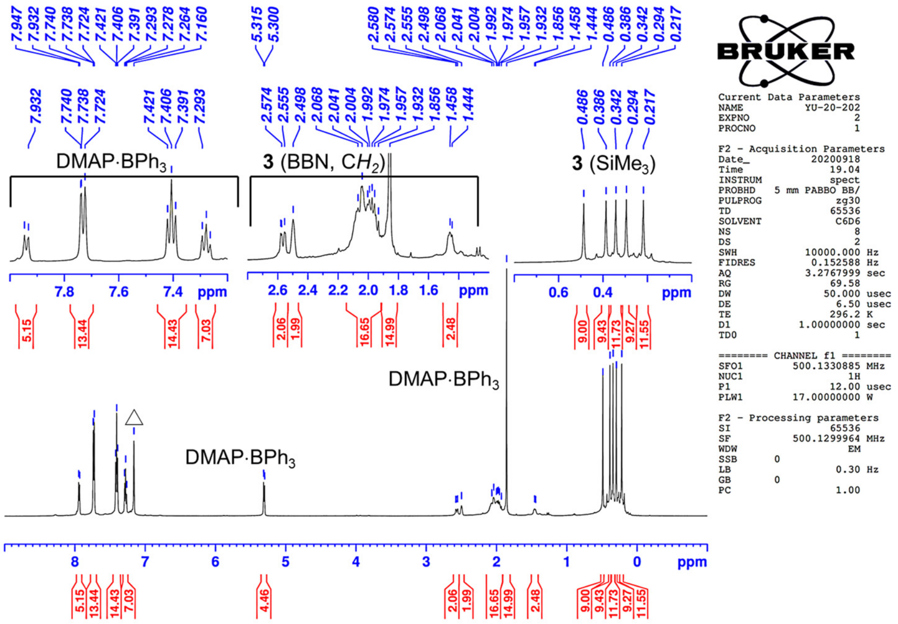

Figure S20. ^1H NMR spectrum of the reaction mixture after addition of $B(C_6H_5)_3$ in C_6D_6 at 296 K (△= C_6D_5H).

Reaction of 5 and Me3SiCl

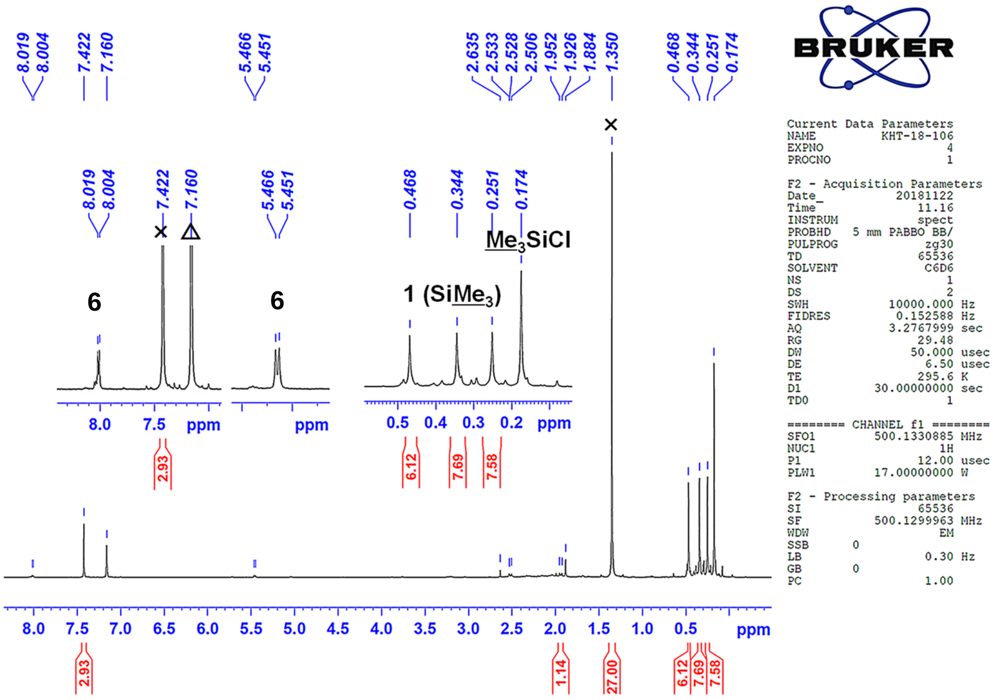

Figure S21. ^{1}H NMR spectrum of reaction mixture of **5** and Me_3SiCl in C_6D_6 at 296 K (△= C_6D_5H, ×=Mes*H).

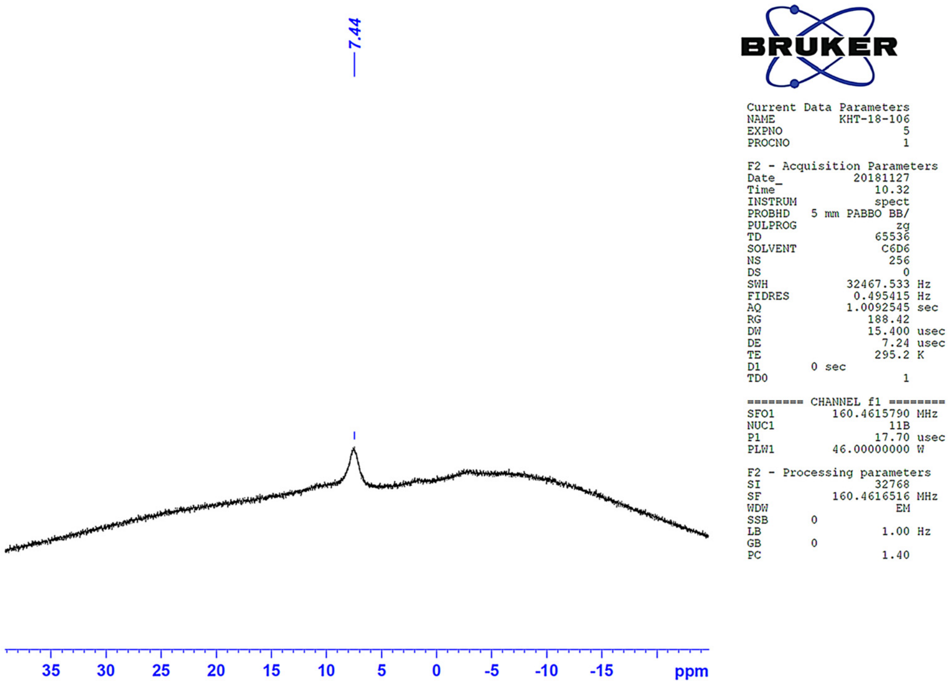

Figure S22. ^{11}B NMR spectrum of reaction mixture of **5** and Me_3SiCl at 295 K.

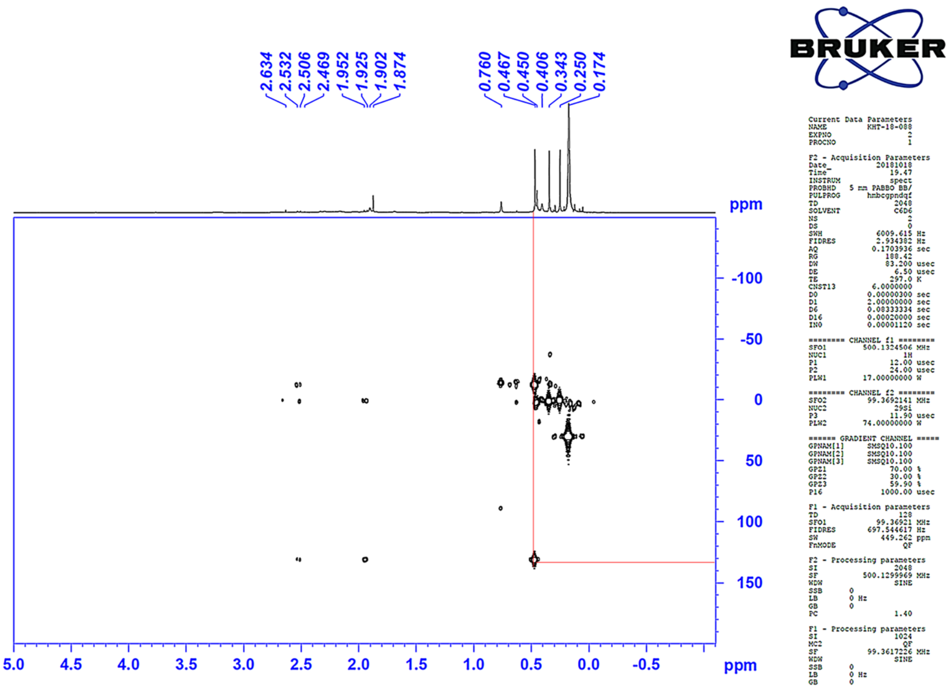

Figure S23. ^{29}Si-^{1}H 2D HMBC NMR spectrum of reaction mixture of **5** and Me_3SiCl in C_6D_6 at 297 K.

Reaction of 4 and DMAP Followed by Me_3SiCl

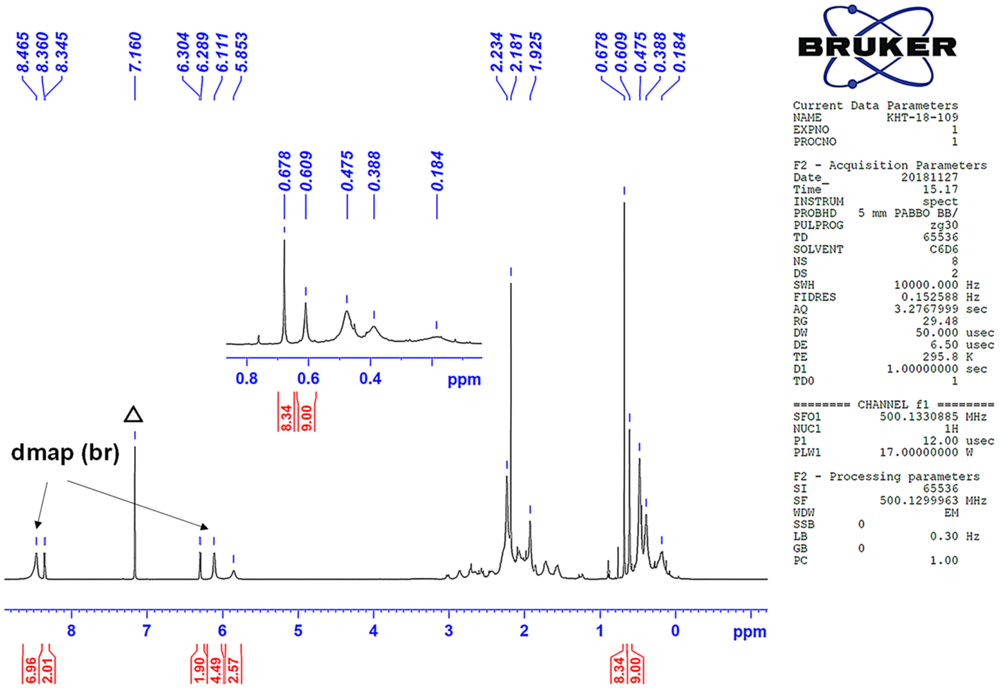

Figure S24. ^{1}H NMR spectrum of the reaction mixture of **4** and DMAP (2 equiv) in C_6D_6 recorded at 296 K (△= C_6D_5H).

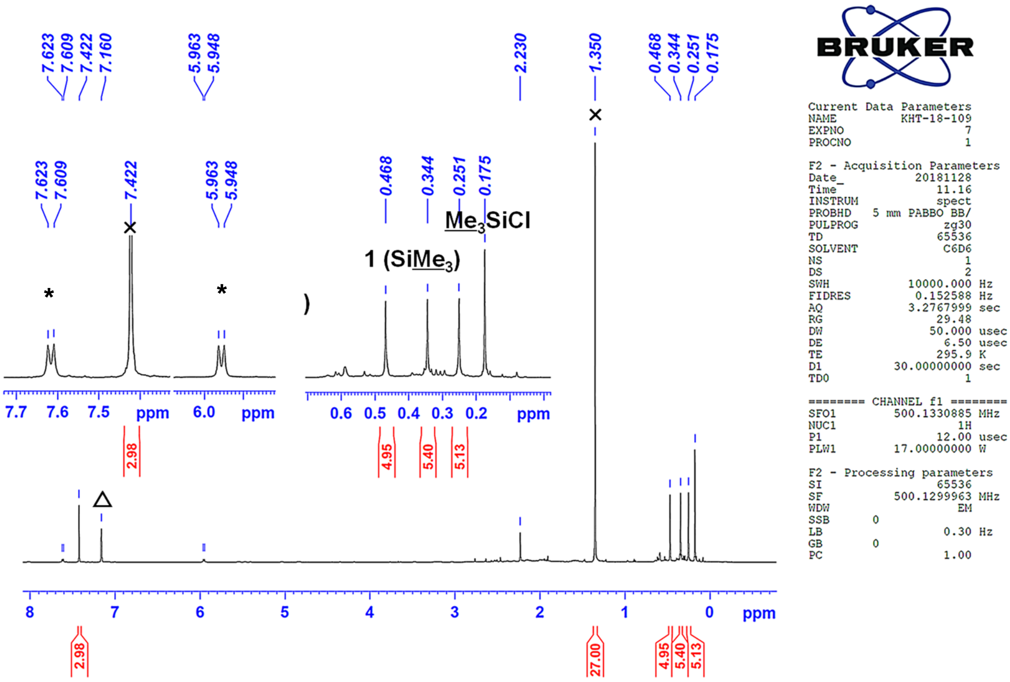

Figure S25. ^{1}H NMR spectrum of the reaction mixture of **4**, DMAP and Me_3SiCl after heating 60 °C in C_6D_6 recorded at 296 K (△= C_6D_5H, ×= Mes*H, * = unidentified byproduct).

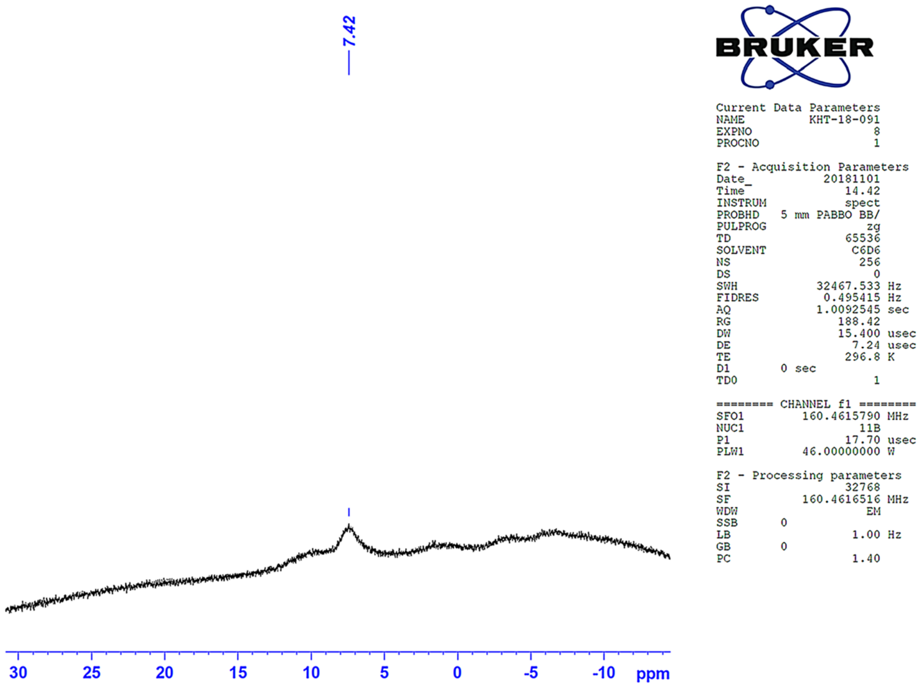

Figure S26. ^{11}B NMR spectrum of reaction mixture of **4**, DMAP and Me_3SiCl after heating 60 °C in C_6D_6 recorded at 297 K.

Reaction of DMAP and BBNCl

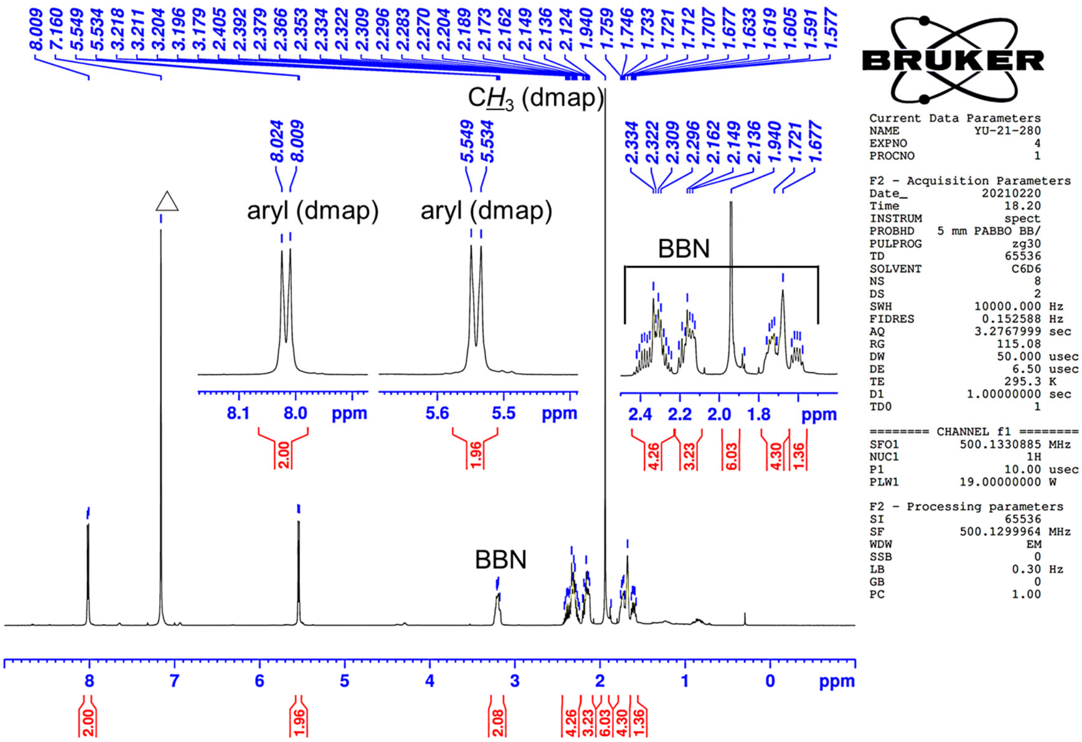

Figure S27. ^{1}H NMR spectrum of **6** in C_6D_6 at 295 K (△= C_6D_5H).

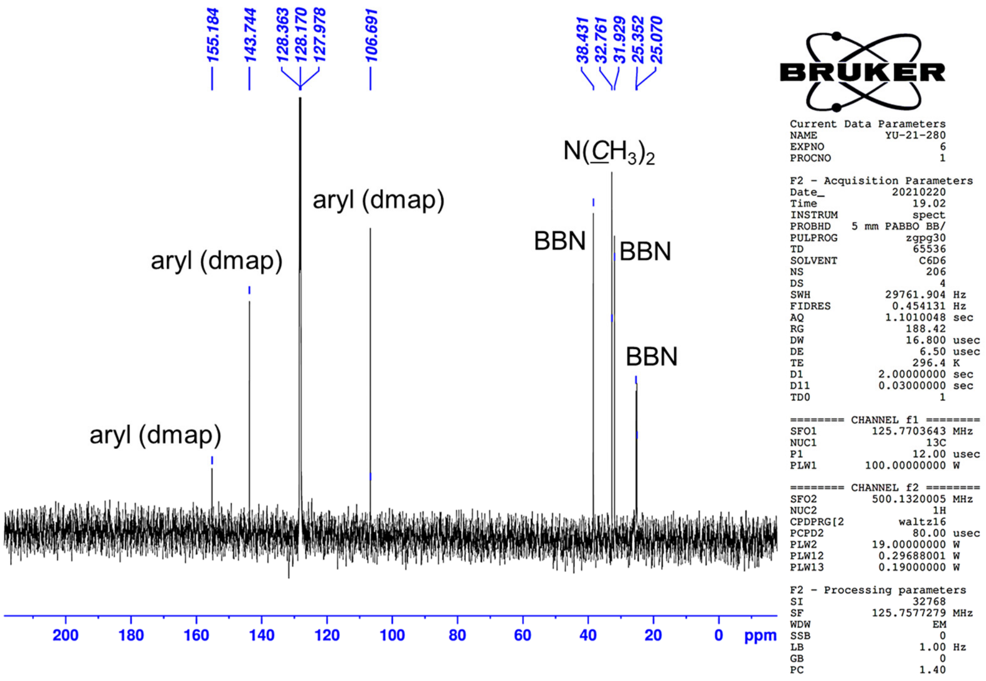

Figure S28. $^{13}C\{^1H\}$ NMR spectrum of **6** in C_6D_6 at 296 K.

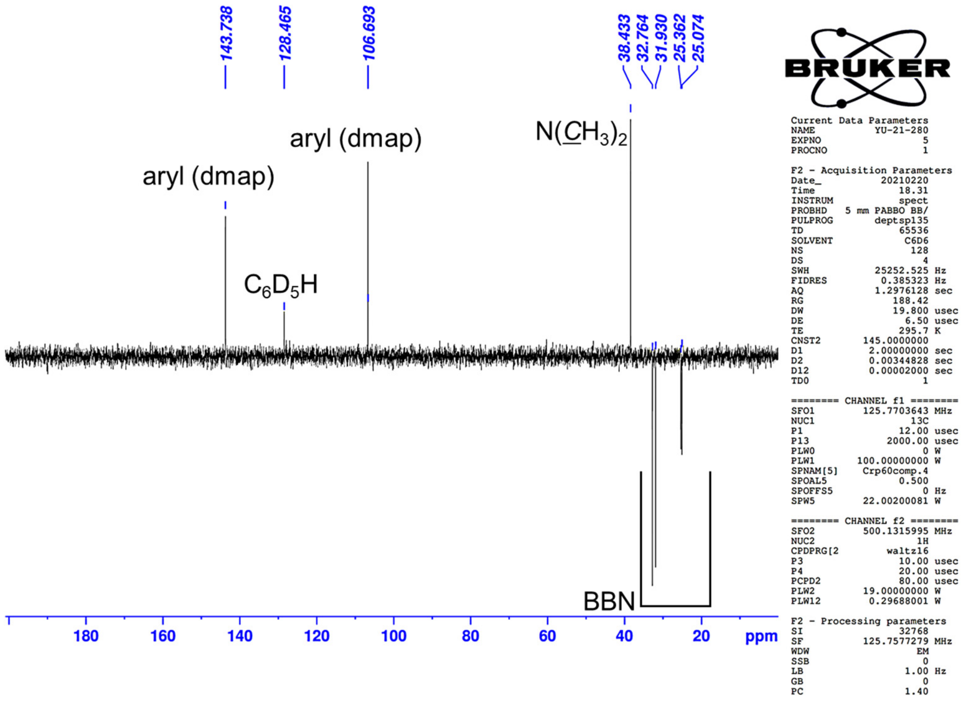

Figure S29. $^{13}C\{^{1}H\}$ NMR spectrum of **6** using DEPT 135 pulse sequence in C_6D_6 at 296 K.

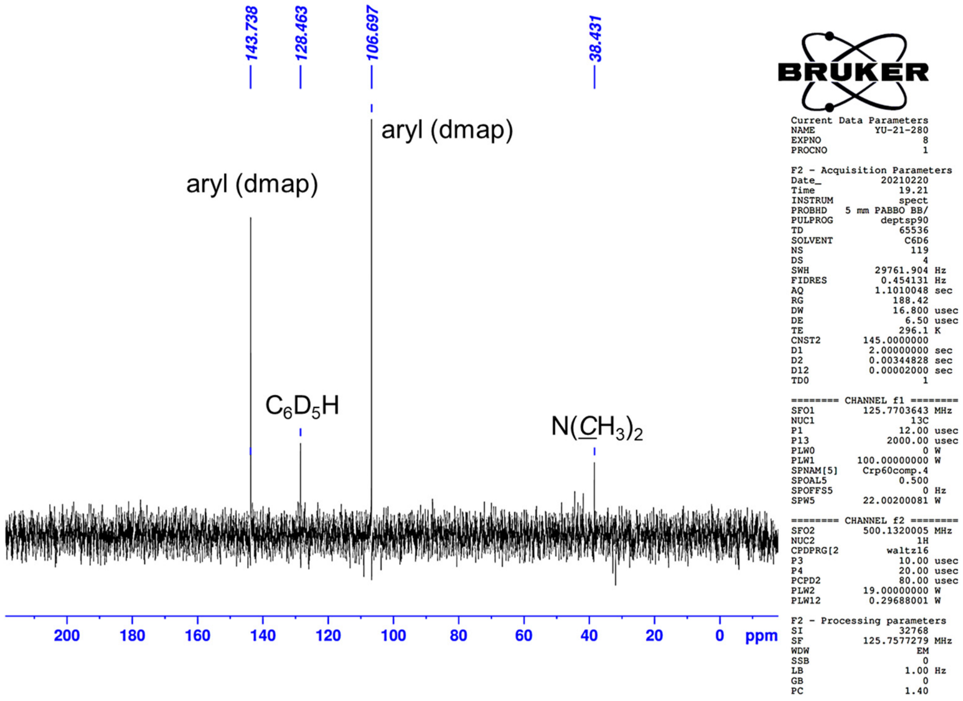

Figure S30. $^{13}C\{^{1}H\}$ NMR spectrum of **6** using DEPT 90 pulse sequence in C_6D_6 at 296 K.

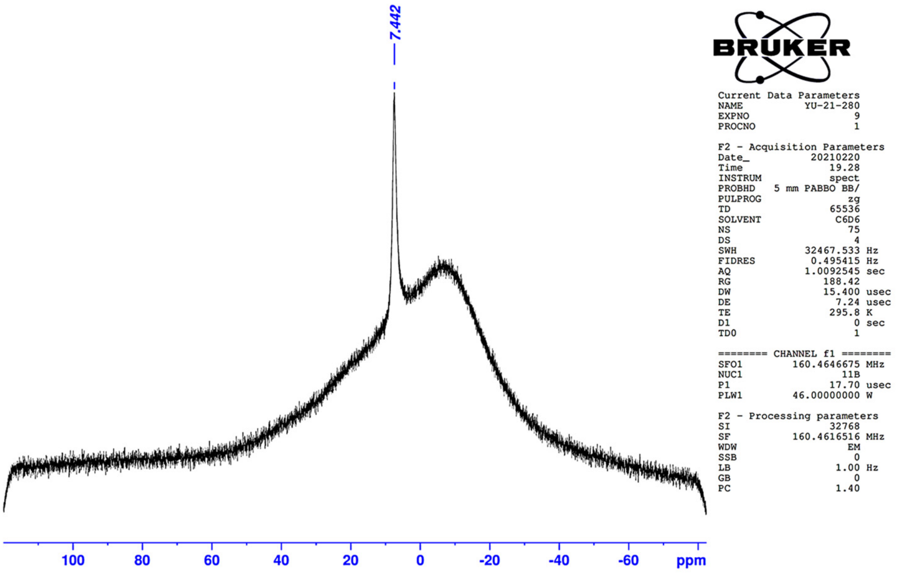

Figure S31. ^{11}B NMR spectrum of **6** in C_6D_6 at 296 K.

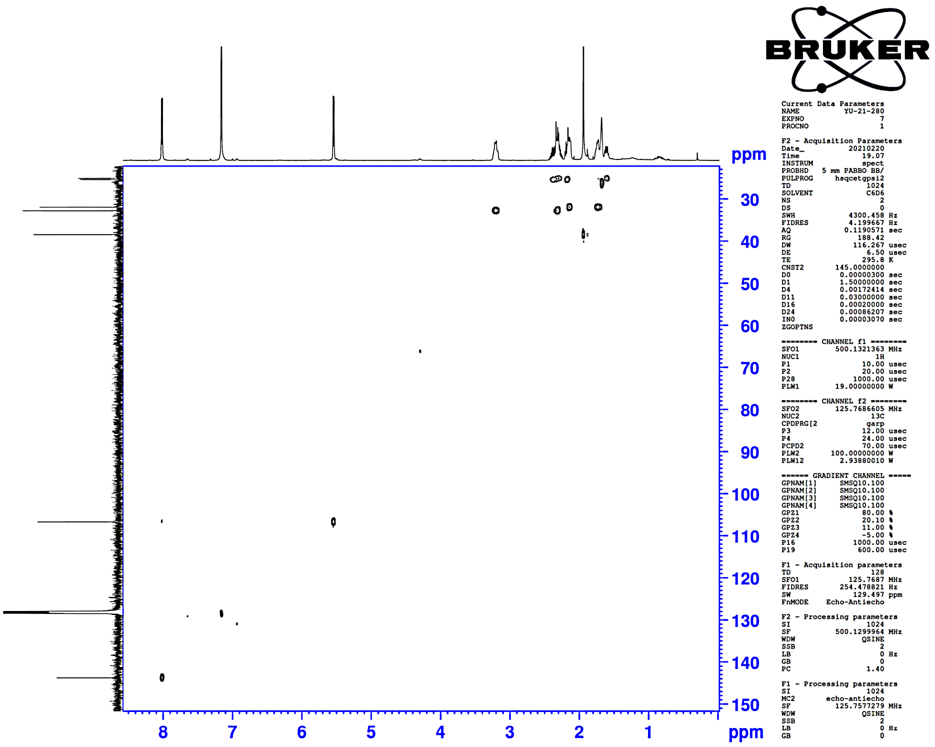

Figure S32. ^{13}C-^{1}H 2D HSQC NMR spectrum of **6** in C_6D_6 at 296 K.

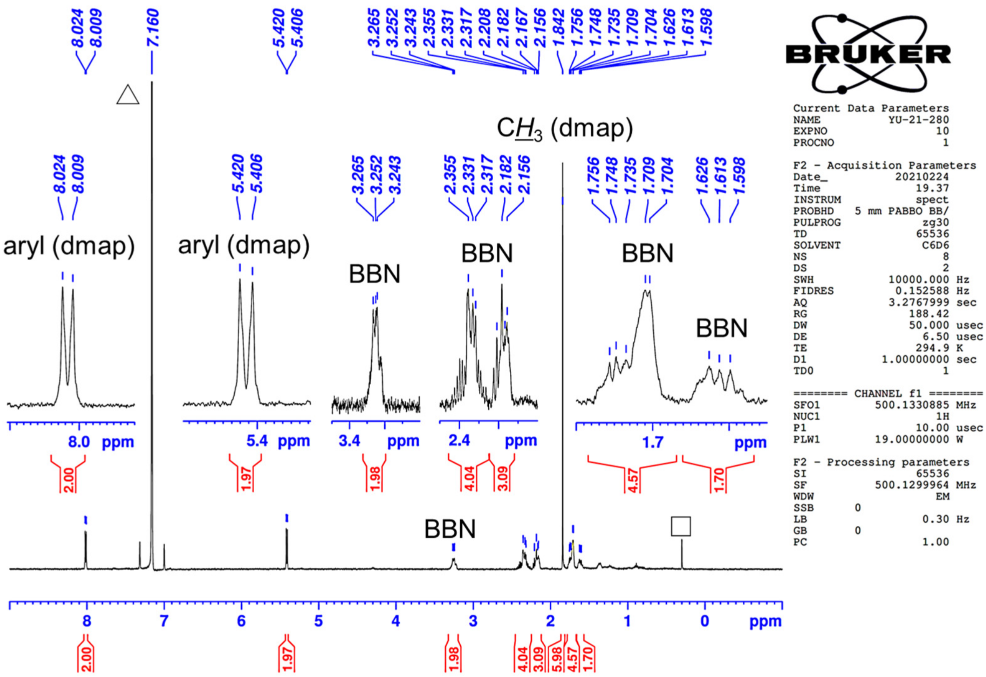

Figure S33. ^{1}H NMR spectrum of **6** in C_6D_6 at 295 K (low concentration, △= C_6D_5H, □ = silicon grease).

2. Details of Theoretical Study

Optimized Structures of 3, 4, and 5

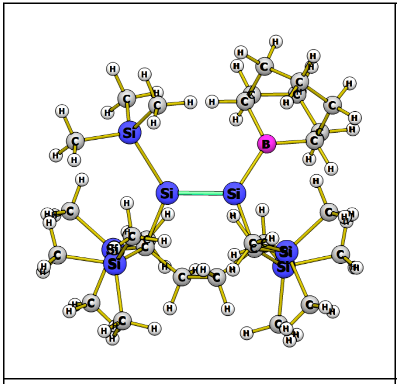

3

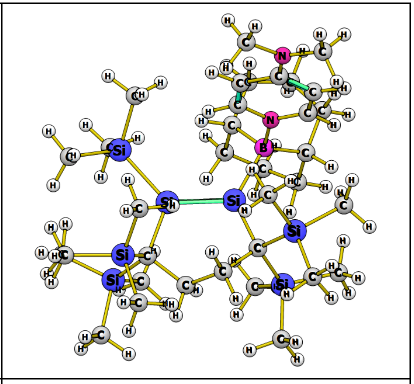

5

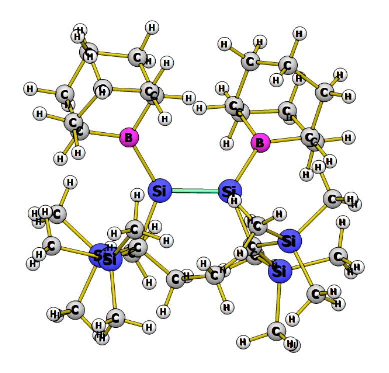

4a

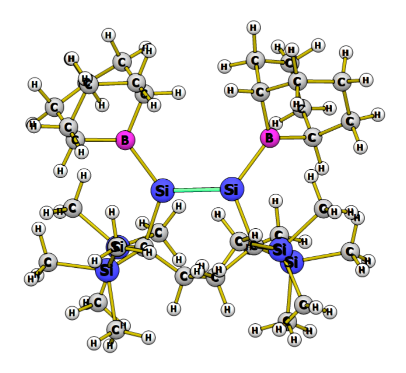

4b

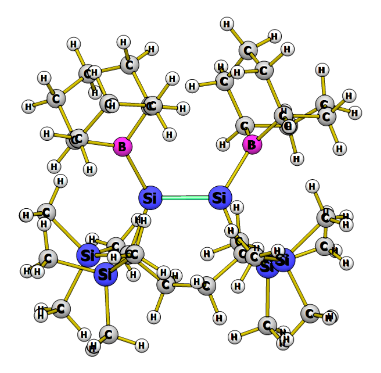

4c

Figure S34. Optimized structures of **3,** three conformers of **4** and **5**.

Table S1. Selected Structural Parameters and Energy Levels of Frontier Orbitals of **3** and **5** Optimized at the B3PW91-D3/6-31(d) Level of Theory

Compound	distance/Å			angle sum/°		twist angle/°		Energy[a]/eV		λ_{max},[a] /nm (f)[a]	JOB
	Si=Si	Si–B	B–N	=Si(–Si)	=Si(–B)	Si=Si	Si–B	HOMO	LUMO		
**3**$_{opt}$	2.19225	1.97039	–	359.10	358.8	20.2	2.5	−4.79	−2.03	487.86 (0.3132)	ti522SiB_2
**3** (solid)	2.1990(8)	1.994(3)	–	359.16(6)	359.15(8)	10.0	5.0	–	–	–	XRD
**3** (solution)	–	–	–	–	–	–	–	–	–	490 (ε 1.9 × 10^4)	in hexane
**5**$_{opt}$	2.19009	2.10907	1.62786	359.69	359.14	8.1	–	−4.06	−0.63 (LUMO+2, π*)	404.4 (0.2108) (π→π*)	ti522SiBDMAP

a) Excitation energy and oscillator strength were calculated at the B3LYP/6-311(2df)[Si], 6-311G(d)[C, B, H] level of theory (solvent = hexane).

Table S2. Selected Structural Parameters and Energy Levels of Frontier Orbitals of **4** Optimized at the B3PW91-D3/6-31(d) Level of Theory

Compound	distance/Å		twist angle			Energy[a]/eV		λ_{max},[a] /nm	ΔG/(kJ/mol)	JOB
	Si=Si	Si–B	Si=Si	Si–B	Si–B	HOMO	LUMO	(f)[a]		
**4a**$_{opt}$	2.20152	1.98758<br>1.99036	27.7	8.3	8.1	−4.90	−2.53	581.07<br>(0.2599)	0.0	ti552B2conf3
**4b**$_{opt}$	2.20285	1.98078<br>2.00371	18.9	13.2	42.6	−4.86	−2.29	539.35<br>(0.3119)	0.34	ti552B2conf1
**4c**$_{opt}$	2.19394	1.96273<br>2.03035	22.2	0.4	72.3	−4.70	−2.08	623.42<br>(0.0506)	11.68	ti552B2conf2
**4**<br>(solid)	2.2114(5)	1.9851(14)<br>2.0156(15)	17.7	15.5	41.2	–	–	–	–	XRD
**4**<br>(solution)	–	–	–	–	–	–	–	576<br>(ε 5260)	–	in hexane

a) Excitation energy and oscillator strength were calculated at the B3LYP/6-311(2df)[Si], 6-311G(d)[C, B, H] level of theory (solvent = hexane).

If the ratio of the molecular number of conformers 2 relative to conformer 1 (N_2/N_1) obeys the Bolzmann distribution, N_2/N_1 should be

$$\frac{N_2}{N_1} = e^{-\frac{E_2 - E_1}{kT}} = e^{-\frac{340\ \mathrm{J/mol} \div 6.02\times10^{23}\,\mathrm{mol}^{-1}}{1.381\ \times\ 10^{-23}\ \mathrm{J\ K^{-1}}\times 298.15\ \mathrm{K}}} = \ e^{-0.137} = 0.872$$

, where Boltzmann constant $k = 1.381 \times 10^{-23}$ J·K^{-1}, Avogadro constant = 6.02×10^{23} mol^{-1}, and temperature T = 298.15 K. This value suggests that both conformers 1 (**4a**) and 2 (**4b**) can be observed.

Natural Bond Orbital (NBO) Analysis

Table S3. NBO Analysis

Compound<br>(R1, R2)	NPA charge				bond order (WBI)<br>[distance/Å]		second order perturbation analysis<br>donor NBO → acceptor NBO,<br>stabilization energy in kcal/mol
	Si1	Si2	B1	B2	Si=Si	Si–B	
**1** ($SiMe_3$, $SiMe_3$)	0.32	0.32	–	–	1.7972<br>[2.17640]	–	–
**3** (BBN, $SiMe_3$)<br>[JOB:ti5552SiB_2_NBO2]	0.43	0.60	0.30	–	1.6581<br>[2.19225]	1.1127<br>[1.97039]	BD2(Si1–Si2)→LV(B2), 25.62
**4** (BBN, BBN)<br>conformer 1<br>[JOB:ti5552B2conf3_NBO2]	0.69	0.67	0.33	0.35	1.5826<br>[2.20152]	1.0647<br>[1.98758]<br>1.0672<br>[1.99036]	BD2(Si1–Si2)→LV(B1), 22.01<br>BD2(Si1–Si2)→LV(B2), 22.03
**4** (BBN, BBN)<br>conformer 2<br>[JOB:ti5552B2conf1_NBO2]	0.64	0.68	0.33	0.38	1.6069<br>[2.20285]	1.0816<br>[1.98078]<br>1.0419<br>[2.00371]	BD2(Si1–Si2)→LV(B1), 22.83<br>BD2(Si1–Si2)→LV(B2), 15.52
**4** (BBN, BBN)<br>conformer 3<br>[JOB:ti5552B2conf2_NBO2]	0.56	0.72	0.28	0.45	1.6121<br>[2.19394]	1.1344<br>[1.96273]<br>0.9765<br>[2.03035]	BD2(Si1–Si2)→LV(B1), 28.56<br>BD2(Si1–Si2)→LV(B2), 0.91
**12** (BBN·DMAP,<br>$SiMe_3$,)	0.74	0.19	0.17	–	1.7721<br>[2.19009]	0.9060<br>[2.10907]	–

GIAO Calculations

Table S4. ^{29}Si NMR Chemical Shifts Calculated at the B97-D3/def2-TZVP Level of Theory

Compound (R1, R2)	Calculated Chemical Shift Relative to TMS (absolute chemical shift)		Observed Chemical Shift Relative to TMS	
	Si-R1	Si-R2	Si-R1	Si-R2
**1** ($SiMe_3$, $SiMe_3$) [JOB:ti5552Si2_ NMR15]	141.4756 (184.4999)	141.9943 (183.9812)	131.4	131.4
**3** (BBN, $SiMe_3$) [JOB:ti5552SiB_2_NMR15]	136.2112 (189.7643)	209.0724 (116.9031)	128.7	187.2
**4** (BBN, BBN) conformer 1 [JOB:ti5552B2conf3_ NMR15]	201.0537 (124.9218)	195.3660 (130.6095)	166.2	166.2
**4** (BBN, BBN) conformer 2 [JOB:ti5552B2conf1_ NMR15]	147.2043 (178.7712)	184.2626 (141.7129)	166.2	166.2
**4** (BBN, BBN) conformer 3 [JOB:ti5552B2conf2_ NMR15]	107.5750 (218.4005)	220.2537 (105.7218)	166.2	166.2
**5** (BBN·DMAP, $SiMe_3$) [JOB:ti5552SiBDMAP_ NMR15]	237.6736 (88.3019)	73.0990 (252.8765)	195.2	87.9
Me_4Si	0 (325.9755)	0 (325.9755)	0	0

TD-DFT Calculations

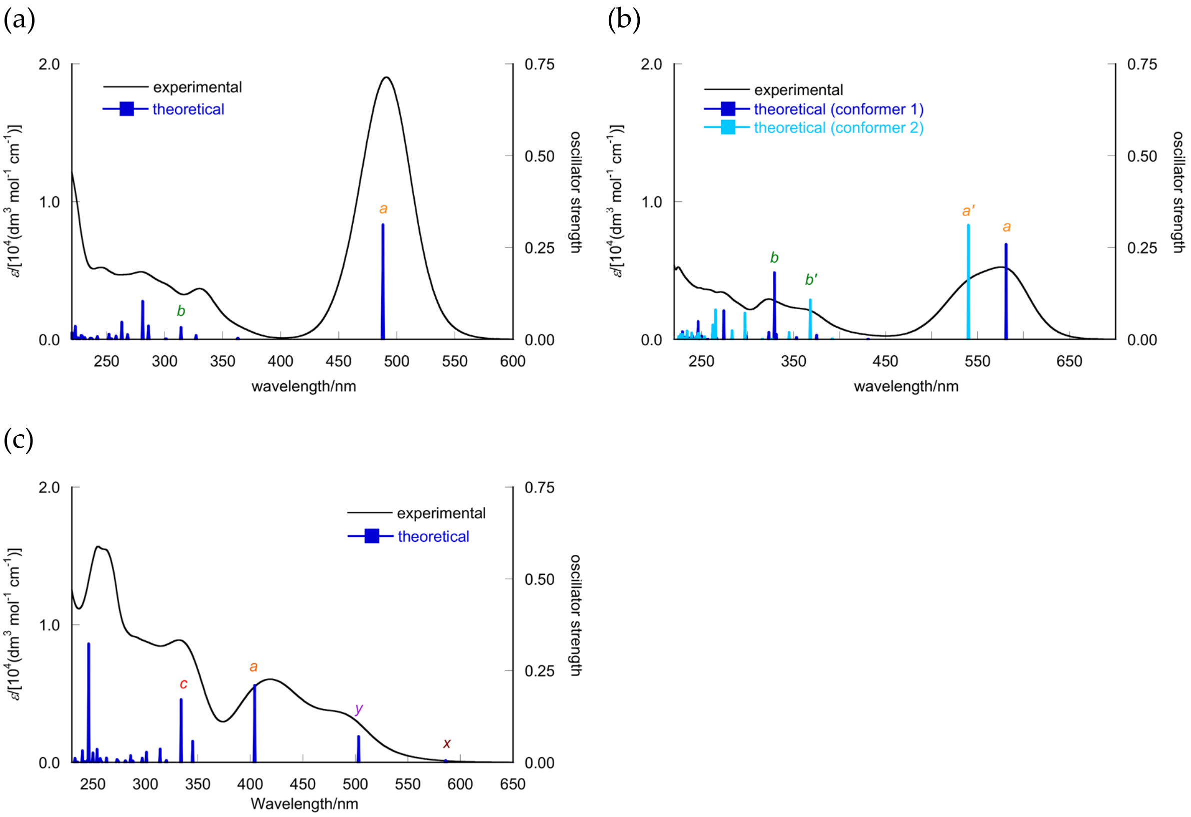

Figure S35. UV-vis absorption spectra of (a) **3**, (b) **4**, and (c) **5** in hexane at room temperature as well as band positions and oscillator strengths (vertical bars) calculated at the B3LYP-D3/B1//B3PW91-D3/6-31G(d) level of theory (B1: 6-311+G(2df) [Si], 6-311G(d) [others]).

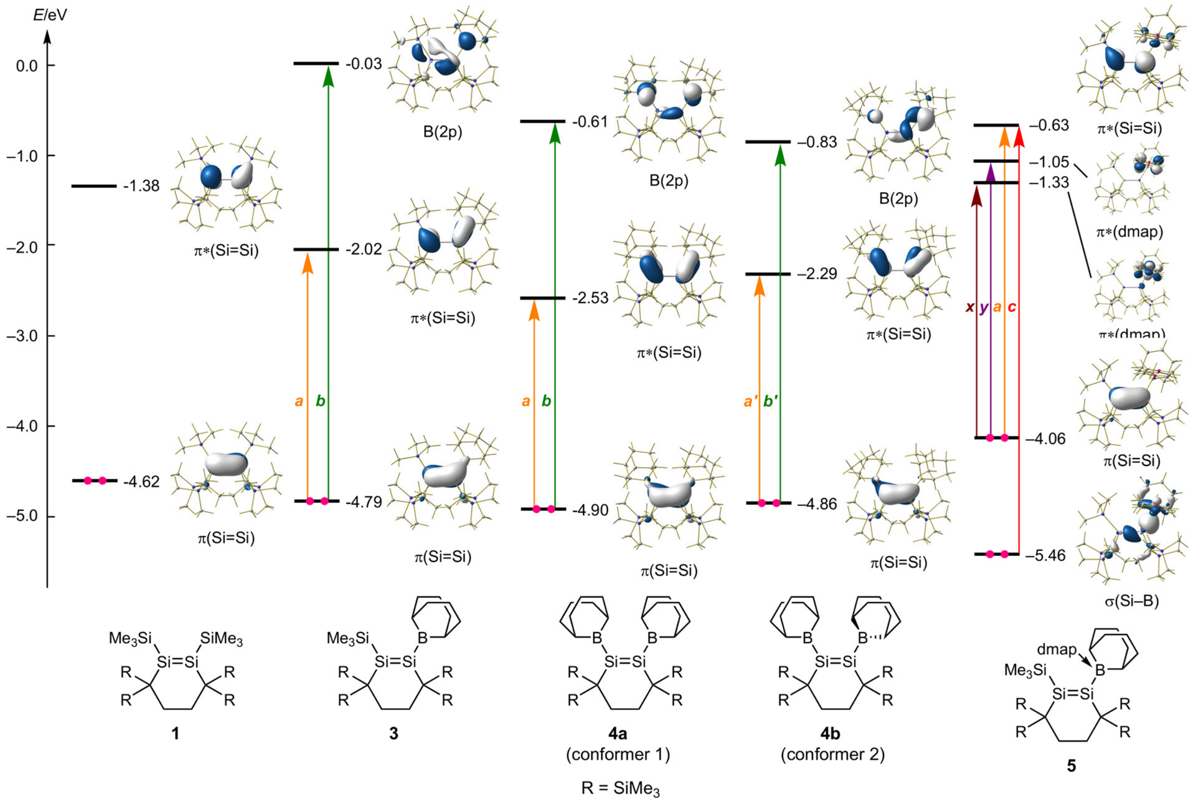

Figure S36. Selected frontier orbitals of $\mathbf{1}_{opt}$, $\mathbf{3}_{opt}$, $\mathbf{4a}_{opt}$, $\mathbf{4b}_{opt}$, and $\mathbf{5}_{opt}$ at the B3LYP-D3/B1//B3PW91-D3/6-31G(d) level of theory (B1: 6-311+G(2df) [Si], 6-311G(d) [others]).

Table S5. Transition Energy, Wavelength, and Oscillator Strengths of the Electronic Transition of **3** Calculated at the TD-B3LYP-D3/B1 [hexane] Level of Theory (The 151st orbital is Highest Occupied π(Si=Si) Orbital Shown in Figure S36.)

```
Excited State   1:      Singlet-A      2.9872 eV  415.04 nm  f=0.2877  <S**2>=0.000
    151 ->152        0.69518
    151 ->153        0.11330

Excited State   2:      Singlet-A      3.5992 eV  344.48 nm  f=0.0313  <S**2>=0.000
    151 ->152       -0.11190
    151 ->153        0.69246

Excited State   3:      Singlet-A      4.1258 eV  300.51 nm  f=0.0030  <S**2>=0.000
    150 ->152        0.69312
    151 ->155        0.10065

Excited State   4:      Singlet-A      4.3196 eV  287.03 nm  f=0.0002  <S**2>=0.000
    151 ->155        0.68555
    151 ->159        0.11591

Excited State   5:      Singlet-A      4.4761 eV  276.99 nm  f=0.0424  <S**2>=0.000
    149 ->152        0.19749
    151 ->154        0.66633
    151 ->156        0.10291

Excited State   6:      Singlet-A      4.5836 eV  270.50 nm  f=0.0111  <S**2>=0.000
    149 ->152        0.62840
    151 ->154       -0.22112
    151 ->156        0.20837

Excited State   7:      Singlet-A      4.7063 eV  263.44 nm  f=0.0194  <S**2>=0.000
    149 ->152       -0.24322
    151 ->156        0.64220
    151 ->161        0.10210

Excited State   8:      Singlet-A      4.7498 eV  261.03 nm  f=0.0028  <S**2>=0.000
    151 ->157        0.64418
    151 ->159        0.24822

Excited State   9:      Singlet-A      5.0169 eV  247.13 nm  f=0.0144  <S**2>=0.000
    147 ->152        0.18348
    148 ->152        0.56741
    151 ->158        0.35633

Excited State  10:      Singlet-A      5.0728 eV  244.41 nm  f=0.0086  <S**2>=0.000
```

```
     147 ->152        -0.16662
     148 ->152        -0.32729
     151 ->158         0.58163
     151 ->159        -0.11517

 Excited State  11:      Singlet-A      5.1184 eV  242.23 nm  f=0.0002  <S**2>=0.000
     151 ->157        -0.26149
     151 ->158         0.12306
     151 ->159         0.63119

 Excited State  12:      Singlet-A      5.1917 eV  238.81 nm  f=0.0060  <S**2>=0.000
     151 ->156         0.12630
     151 ->160         0.68687

 Excited State  13:      Singlet-A      5.2165 eV  237.68 nm  f=0.0156  <S**2>=0.000
     147 ->152         0.65314
     148 ->152        -0.24144

 Excited State  14:      Singlet-A      5.3088 eV  233.55 nm  f=0.0096  <S**2>=0.000
     151 ->156        -0.10846
     151 ->161         0.67346

 Excited State  15:      Singlet-A      5.3830 eV  230.33 nm  f=0.0356  <S**2>=0.000
     150 ->153        -0.26450
     151 ->162         0.60636
     151 ->163        -0.19038

 Excited State  16:      Singlet-A      5.4542 eV  227.32 nm  f=0.0007  <S**2>=0.000
     146 ->152         0.70095

 Excited State  17:      Singlet-A      5.5401 eV  223.79 nm  f=0.0066  <S**2>=0.000
     151 ->162         0.20779
     151 ->163         0.65667

 Excited State  18:      Singlet-A      5.6165 eV  220.75 nm  f=0.0952  <S**2>=0.000
     150 ->153         0.62037
     151 ->162         0.26315

 Excited State  19:      Singlet-A      5.7433 eV  215.88 nm  f=0.0109  <S**2>=0.000
     151 ->161        -0.10249
     151 ->164         0.66117
     151 ->167         0.18322

 Excited State  20:      Singlet-A      5.7879 eV  214.21 nm  f=0.0088  <S**2>=0.000
     151 ->165         0.67678
     151 ->169         0.11657

 Excited State  21:      Singlet-A      5.8762 eV  210.99 nm  f=0.0010  <S**2>=0.000
     143 ->152        -0.12193
     145 ->152         0.66444
     151 ->166        -0.18095

 Excited State  22:      Singlet-A      5.8940 eV  210.36 nm  f=0.0001  <S**2>=0.000
     145 ->152         0.18274
     151 ->166         0.64184
     151 ->169         0.15808

 Excited State  23:      Singlet-A      5.8957 eV  210.30 nm  f=0.0028  <S**2>=0.000
     144 ->152        -0.47087
     151 ->164        -0.12994
     151 ->167         0.47252

 Excited State  24:      Singlet-A      5.9048 eV  209.97 nm  f=0.0108  <S**2>=0.000
     144 ->152         0.45319
     151 ->164        -0.15663
     151 ->167         0.43649
     151 ->168        -0.19860
     151 ->171         0.10422

 Excited State  25:      Singlet-A      5.9386 eV  208.78 nm  f=0.0033  <S**2>=0.000
     144 ->152         0.22448
     151 ->167         0.11550
     151 ->168         0.61327
     151 ->171        -0.17674

 Excited State  26:      Singlet-A      5.9870 eV  207.09 nm  f=0.0013  <S**2>=0.000
     141 ->152         0.10158
     143 ->152         0.66884
     145 ->152         0.11864
     151 ->169        -0.14401

 Excited State  27:      Singlet-A      5.9994 eV  206.66 nm  f=0.0059  <S**2>=0.000
     143 ->152         0.14640
     151 ->165        -0.11510
     151 ->166        -0.16205
     151 ->169         0.63941

 Excited State  28:      Singlet-A      6.0027 eV  206.55 nm  f=0.0645  <S**2>=0.000
     140 ->152         0.11350
     142 ->152        -0.10613
     149 ->153         0.66477

 Excited State  29:      Singlet-A      6.0462 eV  205.06 nm  f=0.0145  <S**2>=0.000
     136 ->152        -0.17057
     139 ->152         0.19405
     140 ->152         0.45616
     142 ->152        -0.42530
     149 ->153        -0.15593

 Excited State  30:      Singlet-A      6.0632 eV  204.49 nm  f=0.0071  <S**2>=0.000
     151 ->168         0.21139
     151 ->171         0.63729

 Excited State  31:      Singlet-A      6.0800 eV  203.92 nm  f=0.0129  <S**2>=0.000
     151 ->170         0.59042
     151 ->172         0.32043

 Excited State  32:      Singlet-A      6.0993 eV  203.28 nm  f=0.0013  <S**2>=0.000
     136 ->152        -0.15067
     140 ->152         0.41682
     142 ->152         0.52167
     144 ->152         0.12136
```

JOB: ti552SiB_2_TD2

Table S6. Transition Energy, Wavelength, and Oscillator Strengths of the Electronic Transition of **4a**$_{\mathbf{opt}}$ Calculated at the TD-B3LYP-D3/B1 [hexane] Level of Theory (The 177th orbital is

Highest Occupied π(Si=Si) Orbital Shown in Figure S36.)

```
Excited State   1:      Singlet-A      2.1337 eV  581.07 nm  f=0.2599  <S**2>=0.000
     177 -> 178        0.71146
     177 <- 178       -0.12290
 This state for optimization and/or second-order correction.
 Total Energy, E(TD-HF/TD-KS) =  -3047.66254997
 Copying the excited state density for this state as the 1-particle RhoCI density.

 Excited State   2:      Singlet-A      2.8777 eV  430.84 nm  f=0.0017  <S**2>=0.000
     176 -> 178        0.70173

 Excited State   3:      Singlet-A      3.3071 eV  374.91 nm  f=0.0124  <S**2>=0.000
     175 -> 178        0.70139

 Excited State   4:      Singlet-A      3.5151 eV  352.72 nm  f=0.0059  <S**2>=0.000
     172 -> 178        0.10087
     174 -> 178        0.68892

 Excited State   5:      Singlet-A      3.7508 eV  330.56 nm  f=0.0157  <S**2>=0.000
     172 -> 178        0.18565
     173 -> 178        0.63195
     177 -> 179       -0.22364

 Excited State   6:      Singlet-A      3.7644 eV  329.36 nm  f=0.1827  <S**2>=0.000
     172 -> 178        0.13670
     173 -> 178        0.19506
     177 -> 179        0.65266

 Excited State   7:      Singlet-A      3.8423 eV  322.68 nm  f=0.0204  <S**2>=0.000
     171 -> 178       -0.16809
     172 -> 178        0.63288
     173 -> 178       -0.20551
     174 -> 178       -0.10623

 Excited State   8:      Singlet-A      4.1620 eV  297.90 nm  f=0.0111  <S**2>=0.000
     171 -> 178        0.68090
     172 -> 178        0.16133

 Excited State   9:      Singlet-A      4.3799 eV  283.08 nm  f=0.0106  <S**2>=0.000
     170 -> 178        0.69037
     177 -> 180       -0.12356

 Excited State  10:      Singlet-A      4.5325 eV  273.55 nm  f=0.0789  <S**2>=0.000
     169 -> 178       -0.19202
     170 -> 178        0.13514
     176 -> 179        0.12049
     177 -> 180        0.62915
     177 -> 181       -0.15256

 Excited State  11:      Singlet-A      4.6525 eV  266.49 nm  f=0.0047  <S**2>=0.000
     169 -> 178        0.62600
     177 -> 180        0.14528
     177 -> 181       -0.18827
     177 -> 184       -0.12111

 Excited State  12:      Singlet-A      4.7037 eV  263.59 nm  f=0.0065  <S**2>=0.000
     168 -> 178        0.66497
     177 -> 181       -0.16138

 Excited State  13:      Singlet-A      4.7277 eV  262.25 nm  f=0.0345  <S**2>=0.000
     168 -> 178        0.21313
     169 -> 178        0.19405
     177 -> 180        0.20451
     177 -> 181        0.54492
     177 -> 183       -0.10355
     177 -> 184        0.17589
     177 -> 185        0.12619

 Excited State  14:      Singlet-A      4.7686 eV  260.00 nm  f=0.0003  <S**2>=0.000
     167 -> 178        0.69624

 Excited State  15:      Singlet-A      4.8317 eV  256.61 nm  f=0.0004  <S**2>=0.000
     166 -> 178        0.68421

 Excited State  16:      Singlet-A      4.8510 eV  255.58 nm  f=0.0013  <S**2>=0.000
     166 -> 178       -0.11080
     176 -> 179        0.66443
     177 -> 180       -0.11440

 Excited State  17:      Singlet-A      4.8993 eV  253.07 nm  f=0.0010  <S**2>=0.000
     165 -> 178        0.69408

 Excited State  18:      Singlet-A      4.9559 eV  250.18 nm  f=0.0098  <S**2>=0.000
     161 -> 178        0.12503
     162 -> 178        0.34147
     163 -> 178        0.40172
     164 -> 178        0.44388

 Excited State  19:      Singlet-A      5.0321 eV  246.39 nm  f=0.0205  <S**2>=0.000
     162 -> 178       -0.13210
     163 -> 178       -0.37308
     164 -> 178        0.45367
     177 -> 181        0.15727
     177 -> 182        0.11375
     177 -> 183        0.19721
     177 -> 184       -0.17384
     177 -> 185       -0.13077

 Excited State  20:      Singlet-A      5.0385 eV  246.07 nm  f=0.0293  <S**2>=0.000
     161 -> 178       -0.11605
     163 -> 178        0.23852
     164 -> 178       -0.25227
     177 -> 181        0.25157
     177 -> 182        0.18397
     177 -> 183        0.32573
     177 -> 184       -0.28641
     177 -> 185       -0.21969

 Excited State  21:      Singlet-A      5.0750 eV  244.30 nm  f=0.0035  <S**2>=0.000
     162 -> 178        0.57242
     163 -> 178       -0.36583
     164 -> 178       -0.13322

 Excited State  22:      Singlet-A      5.1360 eV  241.40 nm  f=0.0007  <S**2>=0.000
     161 -> 178        0.66970
     162 -> 178       -0.12445

 Excited State  23:      Singlet-A      5.1618 eV  240.20 nm  f=0.0097  <S**2>=0.000
     175 -> 179        0.55260
     177 -> 183        0.19448
     177 -> 184        0.28072
     177 -> 186       -0.17877
```

```
 Excited State  24:      Singlet-A      5.1946 eV  238.68 nm  f=0.0005  <S**2>=0.000
     175 -> 179       -0.34005
     177 -> 182        0.34022
     177 -> 183        0.16752
     177 -> 184        0.41579
     177 -> 186       -0.19517

 Excited State  25:      Singlet-A      5.2223 eV  237.41 nm  f=0.0010  <S**2>=0.000
     158 -> 178       -0.15934
     160 -> 178        0.66542

 Excited State  26:      Singlet-A      5.2342 eV  236.87 nm  f=0.0037  <S**2>=0.000
     175 -> 179        0.20927
     177 -> 182        0.55027
     177 -> 183       -0.35613

 Excited State  27:      Singlet-A      5.2769 eV  234.96 nm  f=0.0045  <S**2>=0.000
     177 -> 182        0.12785
     177 -> 183        0.34521
     177 -> 185        0.53086
     177 -> 186        0.23600

 Excited State  28:      Singlet-A      5.3463 eV  231.90 nm  f=0.0071  <S**2>=0.000
     158 -> 178       -0.12754
     159 -> 178        0.67236
     160 -> 178       -0.11587

 Excited State  29:      Singlet-A      5.3993 eV  229.63 nm  f=0.0006  <S**2>=0.000
     158 -> 178        0.44861
     160 -> 178        0.10243
     177 -> 184       -0.16699
     177 -> 185        0.22850
     177 -> 186       -0.42106

 Excited State  30:      Singlet-A      5.4132 eV  229.04 nm  f=0.0164  <S**2>=0.000
     158 -> 178        0.45430
     160 -> 178        0.10047
     174 -> 179        0.27600
     177 -> 184        0.16239
     177 -> 185       -0.14538
     177 -> 186        0.34912

 Excited State  31:      Singlet-A      5.4242 eV  228.57 nm  f=0.0052  <S**2>=0.000
     158 -> 178       -0.18955
     174 -> 179        0.60294
     177 -> 185        0.14357
     177 -> 186       -0.17606

 Excited State  32:      Singlet-A      5.4979 eV  225.51 nm  f=0.0078  <S**2>=0.000
     157 -> 178        0.69062
```

JOB: ti552B2conf3TD2

Table S7. Transition Energy, Wavelength, and Oscillator Strengths of the Electronic Transition of **4b$_{opt}$** Calculated at the TD-B3LYP-D3/B1 [hexane] Level of Theory (The 177th orbital is Highest Occupied π(Si=Si) Orbital Shown in Figure S36.)

```
 Excited State   1:      Singlet-A      2.2988 eV  539.35 nm  f=0.3119  <S**2>=0.000
     177 -> 178        0.70340
     177 <- 178       -0.10341

 Excited State   2:      Singlet-A      3.1749 eV  390.51 nm  f=0.0023  <S**2>=0.000
     176 -> 178        0.70140

 Excited State   3:      Singlet-A      3.3693 eV  367.99 nm  f=0.1086  <S**2>=0.000
     177 -> 179        0.69037

 Excited State   4:      Singlet-A      3.5986 eV  344.54 nm  f=0.0201  <S**2>=0.000
     175 -> 178        0.69622

 Excited State   5:      Singlet-A      3.8080 eV  325.59 nm  f=0.0016  <S**2>=0.000
     173 -> 178        0.16291
     174 -> 178        0.67317

 Excited State   6:      Singlet-A      3.9283 eV  315.61 nm  f=0.0013  <S**2>=0.000
     172 -> 178       -0.13988
     173 -> 178        0.65972
     174 -> 178       -0.15412

 Excited State   7:      Singlet-A      4.1418 eV  299.35 nm  f=0.0001  <S**2>=0.000
     171 -> 178       -0.27680
     172 -> 178        0.61608
     173 -> 178        0.11272

 Excited State   8:      Singlet-A      4.1854 eV  296.23 nm  f=0.0724  <S**2>=0.000
     177 -> 180        0.67055

 Excited State   9:      Singlet-A      4.3874 eV  282.59 nm  f=0.0249  <S**2>=0.000
     171 -> 178        0.63610
     172 -> 178        0.28142

 Excited State  10:      Singlet-A      4.5938 eV  269.89 nm  f=0.0001  <S**2>=0.000
     170 -> 178        0.69429
     176 -> 179       -0.10165

 Excited State  11:      Singlet-A      4.6792 eV  264.97 nm  f=0.0813  <S**2>=0.000
     169 -> 178       -0.14114
     174 -> 179        0.10548
     176 -> 179        0.52707
     177 -> 181        0.36617

 Excited State  12:      Singlet-A      4.7253 eV  262.38 nm  f=0.0405  <S**2>=0.000
     176 -> 179       -0.35174
     177 -> 180       -0.11459
     177 -> 181        0.58229

 Excited State  13:      Singlet-A      4.9026 eV  252.89 nm  f=0.0090  <S**2>=0.000
     169 -> 178        0.68026
     176 -> 179        0.14878
```

```
Excited State  14:      Singlet-A      4.9725 eV  249.34 nm  f=0.0033  <S**2>=0.000
    167 -> 178        -0.31838
    168 -> 178         0.61691

Excited State  15:      Singlet-A      5.0054 eV  247.70 nm  f=0.0029  <S**2>=0.000
    166 -> 178        -0.10891
    167 -> 178         0.60409
    168 -> 178         0.30998

Excited State  16:      Singlet-A      5.0261 eV  246.68 nm  f=0.0168  <S**2>=0.000
    166 -> 178        -0.10553
    174 -> 179        -0.12651
    175 -> 179         0.54060
    176 -> 179         0.14485
    177 -> 182        -0.15984
    177 -> 183         0.26132
    177 -> 184         0.14055

Excited State  17:      Singlet-A      5.0488 eV  245.57 nm  f=0.0169  <S**2>=0.000
    167 -> 178         0.10951
    175 -> 179        -0.34584
    177 -> 182        -0.32851
    177 -> 183         0.38240
    177 -> 184         0.25802
    177 -> 186        -0.10046

Excited State  18:      Singlet-A      5.0851 eV  243.82 nm  f=0.0088  <S**2>=0.000
    166 -> 178        -0.11153
    177 -> 182         0.55342
    177 -> 183         0.18614
    177 -> 184         0.31029
    177 -> 185        -0.11418

Excited State  19:      Singlet-A      5.1061 eV  242.82 nm  f=0.0018  <S**2>=0.000
    163 -> 178         0.10417
    164 -> 178         0.14410
    165 -> 178         0.29054
    166 -> 178         0.57074
    175 -> 179         0.10469
    177 -> 182         0.11369
    177 -> 183         0.10819

Excited State  20:      Singlet-A      5.1409 eV  241.17 nm  f=0.0011  <S**2>=0.000
    164 -> 178         0.30628
    165 -> 178         0.49870
    166 -> 178        -0.35547

Excited State  21:      Singlet-A      5.1824 eV  239.24 nm  f=0.0131  <S**2>=0.000
    162 -> 178         0.11872
    164 -> 178         0.14626
    177 -> 182        -0.12901
    177 -> 183        -0.43451
    177 -> 184         0.46035
    177 -> 185        -0.10402

Excited State  22:      Singlet-A      5.1936 eV  238.73 nm  f=0.0056  <S**2>=0.000
    162 -> 178         0.40086
    163 -> 178        -0.29081
    164 -> 178         0.38274
    165 -> 178        -0.22652
    177 -> 183         0.14759
    177 -> 184        -0.15030

Excited State  23:      Singlet-A      5.2612 eV  235.66 nm  f=0.0015  <S**2>=0.000
    162 -> 178        -0.29460
    163 -> 178         0.36509
    164 -> 178         0.43185
    165 -> 178        -0.29055

Excited State  24:      Singlet-A      5.2980 eV  234.02 nm  f=0.0151  <S**2>=0.000
    162 -> 178        -0.11196
    163 -> 178        -0.25183
    172 -> 179         0.12206
    173 -> 179        -0.12880
    174 -> 179         0.54204
    175 -> 179         0.13774
    177 -> 185         0.15664

Excited State  25:      Singlet-A      5.2984 eV  234.00 nm  f=0.0088  <S**2>=0.000
    162 -> 178         0.44875
    163 -> 178         0.42927
    164 -> 178        -0.15675
    165 -> 178        -0.10161
    174 -> 179         0.19478

Excited State  26:      Singlet-A      5.3361 eV  232.35 nm  f=0.0053  <S**2>=0.000
    161 -> 178        -0.16074
    174 -> 179        -0.23032
    177 -> 184         0.15844
    177 -> 185         0.58371
    177 -> 187         0.16239

Excited State  27:      Singlet-A      5.3620 eV  231.23 nm  f=0.0047  <S**2>=0.000
    161 -> 178         0.66760
    177 -> 185         0.14084

Excited State  28:      Singlet-A      5.4234 eV  228.61 nm  f=0.0150  <S**2>=0.000
    160 -> 178         0.14152
    161 -> 178         0.11250
    172 -> 179        -0.25415
    173 -> 179         0.54395
    174 -> 179         0.17740
    176 -> 180        -0.10709
    177 -> 185         0.11566

Excited State  29:      Singlet-A      5.4528 eV  227.38 nm  f=0.0132  <S**2>=0.000
    173 -> 179        -0.10373
    177 -> 184         0.13771
    177 -> 185         0.10931
    177 -> 186         0.54331
    177 -> 187        -0.32740

Excited State  30:      Singlet-A      5.4621 eV  226.99 nm  f=0.0014  <S**2>=0.000
    158 -> 178        -0.16886
    160 -> 178         0.64203
    173 -> 179        -0.12936
    177 -> 186        -0.12461

Excited State  31:      Singlet-A      5.5171 eV  224.73 nm  f=0.0090  <S**2>=0.000
    177 -> 182        -0.10751
    177 -> 185        -0.17647
    177 -> 186         0.30164
    177 -> 187         0.45194
    177 -> 188         0.22287
    177 -> 189         0.21079
    177 -> 190         0.16525
    177 -> 191        -0.10689

Excited State  32:      Singlet-A      5.5993 eV  221.43 nm  f=0.0023  <S**2>=0.000
    159 -> 178         0.52163
```

```
    171 -> 179       0.13480
    172 -> 179      -0.35329
    173 -> 179      -0.19564
```

JOB: ti552B2conf1TD2

Table S8. Transition Energy, Wavelength, and Oscillator Strengths of the Electronic Transition of **5opt** Calculated at the TD-B3LYP-D3/B1 [hexane] Level of Theory (The 151st orbital is Highest Occupied π(Si=Si) Orbital Shown in Figure S36.)

```
Excited State   1:      Singlet-A      2.1140 eV  586.49 nm  f=0.0058  <S**2>=0.000
    197 -> 198       0.69358
    197 -> 199      -0.12907
 This state for optimization and/or second-order correction.
 Total Energy, E(TD-HF/TD-KS) =  -3501.15742787
 Copying the excited state density for this state as the 1-particle RhoCI density.

 Excited State   2:      Singlet-A      2.4609 eV  503.82 nm  f=0.0715  <S**2>=0.000
    197 -> 198       0.12198
    197 -> 199       0.68452
    197 -> 200      -0.12089

 Excited State   3:      Singlet-A      3.0666 eV  404.30 nm  f=0.2108  <S**2>=0.000
    197 -> 199       0.10605
    197 -> 200       0.67424
    197 -> 202       0.13396

 Excited State   4:      Singlet-A      3.5937 eV  345.01 nm  f=0.0583  <S**2>=0.000
    196 -> 198       0.16015
    197 -> 200      -0.10923
    197 -> 202       0.66304

 Excited State   5:      Singlet-A      3.7123 eV  333.98 nm  f=0.1718  <S**2>=0.000
    196 -> 198       0.66906
    197 -> 202      -0.14751

 Excited State   6:      Singlet-A      3.8748 eV  319.97 nm  f=0.0061  <S**2>=0.000
    196 -> 199       0.67941
    196 -> 200       0.15017

 Excited State   7:      Singlet-A      3.9476 eV  314.08 nm  f=0.0376  <S**2>=0.000
    197 -> 201       0.68234
    197 -> 203      -0.15081

 Excited State   8:      Singlet-A      4.1234 eV  300.68 nm  f=0.0288  <S**2>=0.000
    195 -> 198       0.52892
    195 -> 200       0.10165
    196 -> 199      -0.13298
    196 -> 200       0.39575

 Excited State   9:      Singlet-A      4.1683 eV  297.45 nm  f=0.0124  <S**2>=0.000
    194 -> 198      -0.10991
    195 -> 198      -0.42995
    196 -> 200       0.52358

 Excited State  10:      Singlet-A      4.2977 eV  288.49 nm  f=0.0049  <S**2>=0.000
    197 -> 201       0.14512
    197 -> 203       0.66656
    197 -> 204       0.10600

 Excited State  11:      Singlet-A      4.3420 eV  285.55 nm  f=0.0194  <S**2>=0.000
    195 -> 199      -0.13304
    197 -> 203      -0.10726
    197 -> 204       0.51734
    197 -> 205      -0.36504
    197 -> 206      -0.14692

 Excited State  12:      Singlet-A      4.4096 eV  281.17 nm  f=0.0005  <S**2>=0.000
    195 -> 199      -0.30784
    197 -> 204       0.27080
    197 -> 205       0.43283
    197 -> 206       0.32880

 Excited State  13:      Singlet-A      4.4125 eV  280.99 nm  f=0.0047  <S**2>=0.000
    195 -> 199       0.56336
    195 -> 200       0.13361
    196 -> 200      -0.11727
    197 -> 204       0.30051
    197 -> 205       0.10992
    197 -> 206       0.14620

 Excited State  14:      Singlet-A      4.5238 eV  274.07 nm  f=0.0046  <S**2>=0.000
    194 -> 198       0.63155
    195 -> 198      -0.11546
    195 -> 199       0.15233
    195 -> 200      -0.15831

 Excited State  15:      Singlet-A      4.5368 eV  273.29 nm  f=0.0087  <S**2>=0.000
    194 -> 198       0.10993
    197 -> 204      -0.12245
    197 -> 205      -0.36957
    197 -> 206       0.55217

 Excited State  16:      Singlet-A      4.6767 eV  265.11 nm  f=0.0005  <S**2>=0.000
    194 -> 198       0.19808
    194 -> 199      -0.23005
    195 -> 199      -0.10235
    195 -> 200       0.51865
    197 -> 204       0.11309
    197 -> 207      -0.14168
    197 -> 208      -0.12092
    197 -> 209      -0.19473
    197 -> 212      -0.11520

 Excited State  17:      Singlet-A      4.7159 eV  262.91 nm  f=0.0130  <S**2>=0.000
    194 -> 199      -0.10021
    195 -> 200       0.29928
    197 -> 204      -0.11476
    197 -> 207       0.37131
    197 -> 208       0.22005
    197 -> 209       0.35151
```

```
    197 -> 211       -0.15333
    197 -> 212        0.13954

 Excited State  18:      Singlet-A      4.8045 eV  258.06 nm  f=0.0037  <S**2>=0.000
    197 -> 206        0.11253
    197 -> 207        0.46515
    197 -> 208        0.19301
    197 -> 209       -0.31258
    197 -> 211        0.24218
    197 -> 212       -0.20579
    197 -> 214       -0.11613

 Excited State  19:      Singlet-A      4.8153 eV  257.48 nm  f=0.0113  <S**2>=0.000
    197 -> 207       -0.27980
    197 -> 208        0.54063
    197 -> 210        0.24721
    197 -> 211        0.18314

 Excited State  20:      Singlet-A      4.8402 eV  256.16 nm  f=0.0018  <S**2>=0.000
    194 -> 199        0.62851
    195 -> 199       -0.12786
    195 -> 200        0.23781

 Excited State  21:      Singlet-A      4.8738 eV  254.39 nm  f=0.0367  <S**2>=0.000
    192 -> 198       -0.28834
    193 -> 198        0.57866
    193 -> 199        0.18999

 Excited State  22:      Singlet-A      4.9506 eV  250.44 nm  f=0.0198  <S**2>=0.000
    192 -> 199        0.35866
    193 -> 198       -0.13959
    193 -> 199        0.53231
    194 -> 199       -0.10076
    197 -> 212        0.10879

 Excited State  23:      Singlet-A      4.9603 eV  249.95 nm  f=0.0072  <S**2>=0.000
    197 -> 208       -0.21632
    197 -> 209        0.42314
    197 -> 210        0.19789
    197 -> 211        0.22127
    197 -> 212       -0.36541
    197 -> 213       -0.12152

 Excited State  24:      Singlet-A      4.9838 eV  248.78 nm  f=0.0022  <S**2>=0.000
    197 -> 208       -0.14566
    197 -> 211        0.45586
    197 -> 212        0.36576
    197 -> 213        0.28282

 Excited State  25:      Singlet-A      5.0454 eV  245.74 nm  f=0.3240  <S**2>=0.000
    192 -> 198        0.59862
    193 -> 198        0.29684
    197 -> 210       -0.10590

 Excited State  26:      Singlet-A      5.0573 eV  245.16 nm  f=0.0075  <S**2>=0.000
    192 -> 198        0.12329
    197 -> 207        0.12400
    197 -> 208       -0.13630
    197 -> 210        0.56827
    197 -> 211       -0.21441
    197 -> 212        0.19195
    197 -> 214       -0.14416

 Excited State  27:      Singlet-A      5.1163 eV  242.33 nm  f=0.0038  <S**2>=0.000
    191 -> 198        0.13238
    192 -> 199        0.36729
    193 -> 199       -0.19644
    193 -> 200       -0.13288
    194 -> 200        0.47823
    197 -> 212       -0.10056

 Excited State  28:      Singlet-A      5.1586 eV  240.34 nm  f=0.0174  <S**2>=0.000
    192 -> 199       -0.17224
    193 -> 199        0.11591
    197 -> 210        0.12575
    197 -> 212       -0.21174
    197 -> 213        0.50748
    197 -> 214        0.31054

 Excited State  29:      Singlet-A      5.1631 eV  240.14 nm  f=0.0018  <S**2>=0.000
    192 -> 199       -0.34128
    192 -> 200       -0.10490
    193 -> 199        0.24454
    193 -> 200        0.19734
    194 -> 200        0.47837
    197 -> 213       -0.13122

 Excited State  30:      Singlet-A      5.1753 eV  239.57 nm  f=0.0124  <S**2>=0.000
    190 -> 198       -0.14763
    191 -> 198        0.66104
    194 -> 200       -0.11909

 Excited State  31:      Singlet-A      5.2703 eV  235.25 nm  f=0.0020  <S**2>=0.000
    196 -> 202        0.11010
    197 -> 210        0.14437
    197 -> 211        0.17634
    197 -> 212        0.12675
    197 -> 213       -0.29868
    197 -> 214        0.54246
    197 -> 217       -0.10251

 Excited State  32:      Singlet-A      5.3287 eV  232.67 nm  f=0.0119  <S**2>=0.000
    196 -> 202        0.39779
    197 -> 216        0.50316
    197 -> 219       -0.18118
```

JOB: ti552SiBDMAP_TD2

3. Temperature-Dependent UV-vis Spectrum of 4

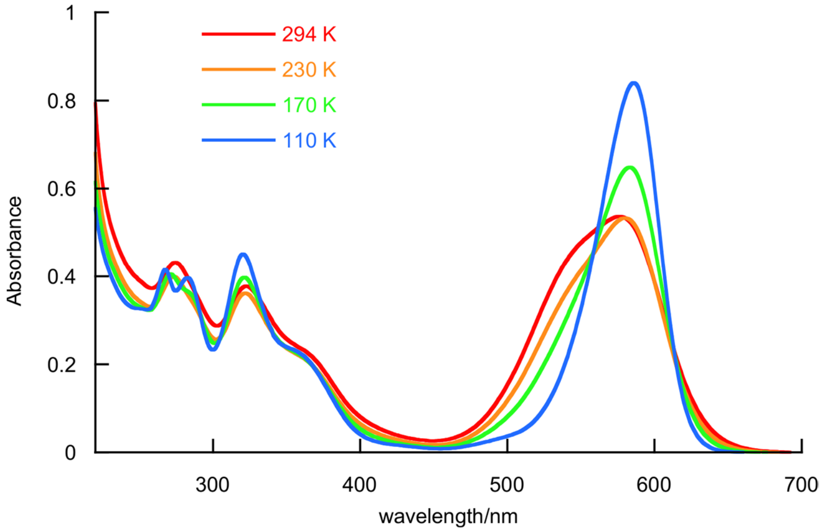

Figure S36. UV-vis absorption spectrum **4** at various temperatures in 3-methylpentane.
